# Beyond bisphosphonates: radiolabeled phosphopeptides for bone targeting

**DOI:** 10.1186/s13550-026-01437-5

**Published:** 2026-04-26

**Authors:** Mazen Jamous, Barbara Roether, Eric Mühlberg, Christian Kleist, Armin Kübelbeck, Uwe Haberkorn, Walter Mier

**Affiliations:** 1 Department of Nuclear Medicine, Heidelberg University Hospital, Im Neuenheimer Feld 400, 69120 Heidelberg, Germany; 2 Department of Pharmaceutical Technology and Biopharmacy, Heidelberg University, Im Neuenheimer Feld 329, 69120 Heidelberg, Germany

**Keywords:** Bone targeting, Bisphosphonates, Hydroxyapatite, Phosphopeptides

## Abstract

**Background:**

The efficacy of current bone-targeting agents, notably bisphosphonates, in the treatment of bone metastases remains limited by their systemic toxicity and excessively long half-life. This study aims to develop bone-targeting agents inspired by osteotropic peptides involved in the bone mineralization process. These agents are intended to provide an innovative alternative to bisphosphonates for precision bone targeting.

**Results:**

Osteotropic peptides and phosphopeptides were obtained by solid-phase synthesis and conjugated to DOTA. The peptides were radiolabeled with gallium-68 or lutetium-177, and their binding affinity to bone was tested in vivo. Osteopontin and matrix extracellular phosphoglycoprotein (MEPE) derived peptides did not show strong binding to bone. Systematic variations in oligoglutamic acid chain length, as well as the positioning and clustering of phosphorylated serine residues, enabled the identification of an optimized phosphopeptide. Clustering phosphorylated sites within the peptide sequence provided significant advantages over phosphorylated moieties scattered throughout the peptide sequence. DOTA-pS_4_E_8_ showed the strongest affinity for bone, comparable to the clinically used bone targeting agent methylene bisphosphonate (MBP).

**Conclusions:**

The novel phosphopeptides match the outstanding bone-targeting capabilities of bisphosphonates and show comparable pharmacokinetics. Owing to their peptidic nature and the consequently anticipated favorable toxicological profile, these agents warrant further investigation as versatile bone-targeting vectors.

**Graphical Abstract:**

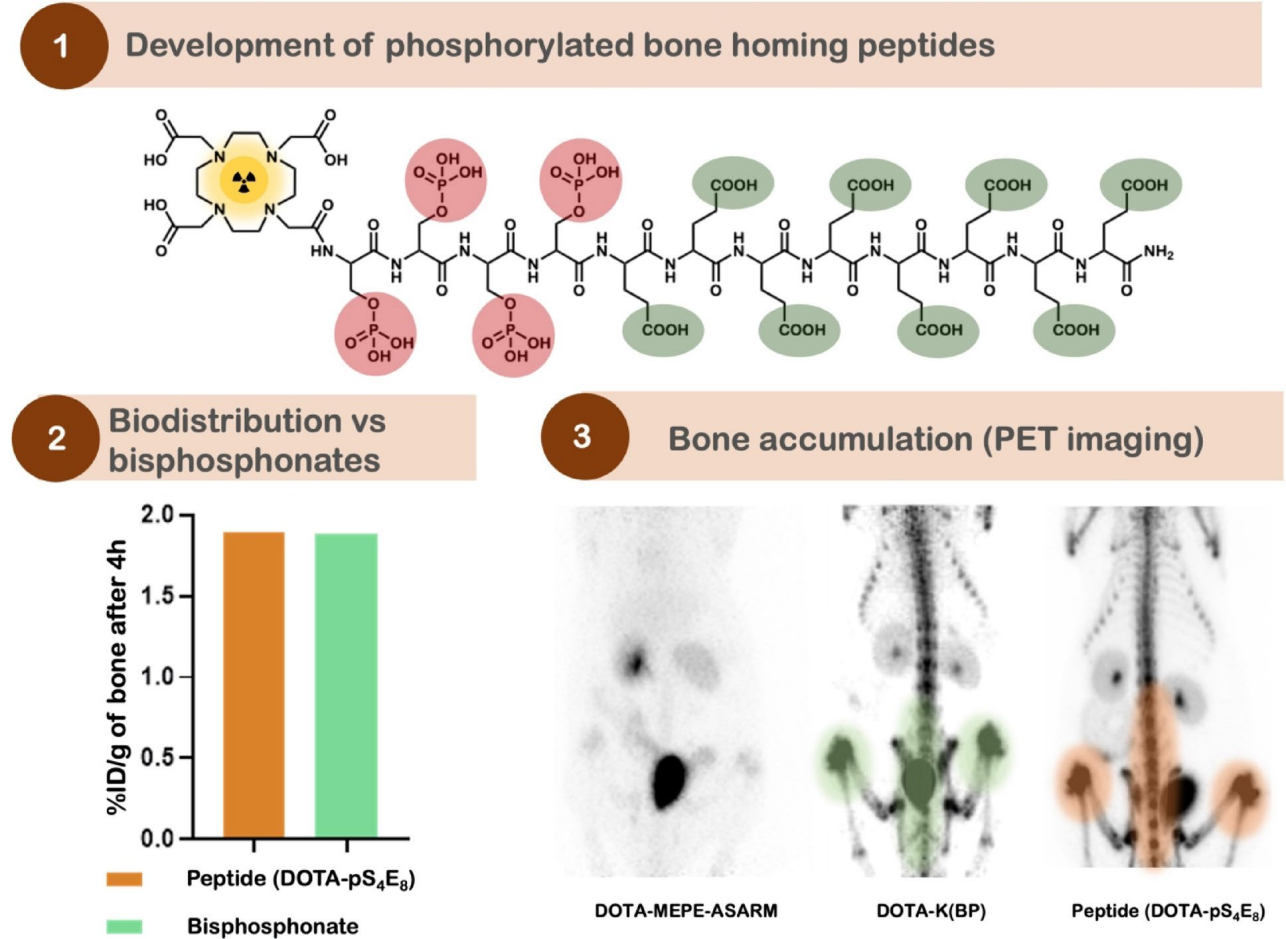

**Supplementary Information:**

The online version contains supplementary material available at 10.1186/s13550-026-01437-5.

## Background

 Targeted therapies have revolutionized cancer treatment. Nuclear medicine approaches excel here, as they enable the use of ultra-low drug quantities – critical for minimizing receptor saturation [1]. However, the range of clinically viable tumor targets, such as somatostatin receptors (SSTRs) and prostate-specific membrane antigen (PSMA), is limited. Broadening the spectrum of highly specific and therapeutically relevant tumor targets is now a central goal, yet advances comparable to those seen with fibroblast activation protein (FAP) are rare. Given that the key distinctions between tumor and healthy tissue have been known for years and extensively explored for tracer development, the likelihood of revolutionary discoveries continues to diminish. Conversely, physiological targeting mechanisms (e.g., hypoxia-targeting agents, lymphatic system targeting tracers, cell adhesion molecules, or bone-seeking compounds that accumulate preferentially in metastatic niches) remain underexplored despite their potential for tissue- or pathology-specific therapeutic precision.

Over the last 40 years, bisphosphonates have become the most established bone-targeting agents for therapy and diagnostics of bone diseases. Their specific binding to farnesyl pyrophosphate synthase and complexation of Ca^2+^ ions in hydroxyapatite leads to bone accumulation, in particular in high metabolic epiphyseal plates and other joint regions [2–4]. Due to the increased activity of farnesyl pyrophosphate synthase, bisphosphonate-based tracers attain excellent efficiency in the specific targeting of metastatic bone disease [5, 6]. Bisphosphonates are exceptionally resistant to chemical and enzymatic degradation, resulting in in vivo half-lives reaching several years [7]. While this is advantageous for their therapeutic use to inhibit bone resorption, it presents a disadvantage for their use as a targeting moiety. PSMA-targeting tracers like [^68^Ga]Ga-PSMA-11 and [^18^F]F-PSMA have also shown a high sensitivity and specificity in detecting bone metastases. However, there is a notable rate of false-positive results, necessitating a careful interpretation of the image [8, 9]. Consequently, efforts have been made to develop alternative targeting moieties. The most efficient ones are based on acidic peptides [10]. However, the performance of the known peptides still lags strongly behind bisphosphonates [7]. In this study, we were able to show that the incorporation of phosphate groups into the sequence of bone-targeting peptides strongly augments their affinity. The rational design of this novel class of tracers leads to compounds that match the bone-targeting specificity of the gold standard bisphosphonates.

Bone comprises an organic component and an inorganic bone mineral component in the form of small crystals. It is composed of approximately 50–70% mineral, 20–40% organic extracellular matrix, 5–10% water, and 1–5% lipids. Hydroxyapatite, a calcium phosphate mineral with the approximate formula Ca_10_(PO_4_)_6_(OH)_2_, is the primary inorganic component responsible for the solidity and mechanical strength of bone. It is organized in long, needle-like crystals embedded in collagen fibrils. Hydroxyapatite is associated with many pathological changes in the bone. This unique property led to its use as a biomarker for target molecules for the selective bone delivery of therapeutics to treat bone-related pathologies [7]. Radiolabeled bisphosphonates have been developed for the imaging of bone disorders and for their respective therapeutic purposes. Depending on the purpose, [^68^Ga] and [^99m^Tc] are the preferred nuclides for diagnostic applications, and [^177^Lu] and [^90^Y] are for therapeutic applications [11]. Bisphosphonates, such as MBP (methylene bisphosphonate) and EDTMP (ethylenediamine tetra(methylene phosphonic acid)), are acyclic phosphonic acids that serve dual functions: they form a complex with a radionuclide and they adsorb to hydroxyapatite, thus enabling diagnosis and therapeutic approaches. Bisphosphonates with an added chelator such as [^177^Lu]Lu-BPAMD, [^177^Lu]Lu-DOTA^ZOL^, [^177^Lu]Lu-DOTA-IBA, [^68^Ga]Ga-THP-Pam, and [^68^Ga]Ga/[^177^Lu]Lu-P15-041 are also widely used and show promising results in enhancing targeting accuracy [3, 12–17].

In routine clinical use, [^18^F] sodium fluoride and bisphosphonate-based radiopharmaceuticals (such as MBP) are utilized. They act as diagnostic agents or palliative agents in the treatment of pain in bone diseases. Beyond these agents, calcium-mimetic radionuclides such as [^223^Ra]RaCl_2_ (Xofigo^®^) and [^89^Sr]SrCl_2_ (Metastron^®^) are used to deliver localized beta or alpha radiation to sites of increased bone remodeling activity for palliative or survival-prolonging therapy in metastatic bone disease. However, both agents are associated with dose-limiting hematologic toxicity [18, 19]. Bisphosphonates are the prototype of bone-targeting drugs, providing stable bone-tracing molecules for the treatment of metabolic bone diseases. As a major side effect of bisphosphonate treatment, patients suffer from osteonecrosis of the jaw and extra-and skeletal adverse effects. This treatment can cause gastrointestinal problems such as nausea, abdominal fullness, epigastric pain, and hypocalcaemia due to the calcium-binding activity of bisphosphonates [7].

Early studies of bone matrix proteins identified one family of non-collagenous proteins of the small integrin-binding ligand *N*-linked glycoprotein (SIBLING family) with high affinity to hydroxyapatite. These hydroxyapatite mineral-binding proteins regulate the hydroxyapatite mineralization within the matrix. The SIBLING family includes bone sialoprotein (BSP), dentin matrix protein 1 (DMP1), matrix extracellular phosphoglycoprotein (MEPE), osteopontin (OPN), and dentin sialophosphoprotein (DSPP). The common property among all these proteins is their affinity to calcium ions through negatively charged groups, e.g. phosphate and carboxylate. For example, the phosphoprotein osteopontin interacts with calcium ions through aspartic acid-rich domains and its negatively charged phosphorylated serine residues. There has been extensive research on MEPE, and an acidic serine- and aspartic-rich motif (ASARM) peptide as a functional domain has been identified. This motif is highly conserved in other SIBLING proteins as well. Other structural features of SIBLING proteins are the post-translational modifications, such as phosphorylation and glycosylation, that can be important for their function in the bone. Matrix extracellular phosphoglycoprotein (MEPE) is a phosphoglycoprotein (45 kDa) that inhibits the mineralization of bone [20]. The inhibitory effect was localized to a small carboxyl-terminal MEPE peptide, which is released by cathepsin B and contains an acidic serine-aspartate-rich motif (MEPE-ASARM) [20, 21]. In addition, the peptide is phosphorylated at three serine residues, which increases the negative overall charge of the peptide and, therefore, improves its binding affinity to hydroxyapatite. Osteopontin (OPN) is a secreted 44 kDa phosphoprotein, with the rat bone OPN containing 301 amino acids. The substance contains high levels of aspartic acid, glutamic acid, and serine residues, which, similar to threonine residues, are phosphorylated. OPN also exerts a variety of effects in the mineralization process in vitro [22]. The effects of this process vary depending on the position of the crystal and the extent of its phosphorylation. In some cases, the effects support the formation and growth of hydroxyapatite, while in other cases, they inhibit mineralization.

## Methods

The reagents and solvents were obtained from Merck (Darmstadt, Germany) and used without purification steps. Amino acid building blocks were from Novabiochem (Bad Soden, Germany). Cyclen was purchased from Strem Chemicals (Karlsruhe, Germany). DOTA was synthesized as described [23–25]. RP-HPLC analyses were carried out on C-18 columns with a linear gradient from water to acetonitrile, both containing 0.1% TFA. Chromatograms were recorded on an Agilent 1100 series HPLC system supplying a flow of 2 mL/min in 5 min with a Merck Chromolith RP-18e column (100 × 3 mm). Semi-preparative purifications were performed using a Gilson 321 HPLC. The column applied was a Merck Chromolith RP-18e (100 × 10 mm). The peptide conjugates were characterized using ESI mass spectrometry by an Orbitrap Technology (Exactive, Thermo Fisher Scientific, Waltham, MA, USA) connected to an Agilent 1200 HPLC system with a Hypersil Gold C18, 1.9 mm column (2.1 × 200 mm). Mass spectra were obtained by scanning from m/z = 200–4000 for 30 min. HPLC conditions: a linear gradient of 0.05% TFA in water to 0.05% TFA in acetonitrile in 30 min, flow of 0.2 mL/min, 60 °C, absorbance λ = 214 nm.

### Solid-phase peptide phosphopeptide synthesis

The peptide substrates with unprotected serine or tyrosine residues were assembled on a fully automated synthesizer via solid-phase peptide synthesis using the Fmoc strategy on a TentaGel resin [26]. The protected chelator was coupled to the *N*-terminus of the peptide using HATU as an activating agent. Then, a post-synthetic phosphorylation step was performed using dibenzyl-*N*,*N*-diisopropylphosphoramidite. The deprotection of the benzyl and other protecting groups and the cleavage of the peptide were performed simultaneously with TFA [25, 27]. The phosphopeptides were purified by preparative HPLC using a Gilson 321 pump HPLC system. Lyophilization afforded the products as white solids, with yields varying from 20% to 25%. Characterization by RP-HPLC and ESI/MS, confirmed 90% purity for all compounds (Supplemental Fig. 7–20).

### In vivo small animal pet imaging

All animal experiments were conducted in female Wistar rats (200–250 g, 6–8 weeks) or NMRI mice (25–30 g, 4–5 weeks), sourced from Charles River Laboratories, acclimatized for one week, and housed on a 12:12 h light-dark cycle with ad libitum access to standard chow and water. For Positron emission tomography (PET) measurements the rats were anesthetized with sevoflurane (2 vol%) (Abbott, Wiesbaden, Germany) and O_2_ (0.5 l/min), intravenously injected into the tail vein and scanned in a prone position on a Siemens Inveon micro-PET scanner (Siemens, Knoxville, TN, USA) at 1 h and 2 h (transmission 15 min; emission 20 min) after injection of the respective ^68^Ga-labeled conjugate (approx. 1 MBq, 20 GBq/µmol). PET images were reconstructed with OSSEM 3D MAP using the Inveon Research Workplace system software (Siemens, Knoxville, TN, USA).

Further details on characterization, synthesis, radiolabeling, and molar/injected activity of the compounds are provided in the Supplementary Information and Table 1.

### Biodistribution studies

Biodistribution studies of radiolabeled peptides and phosphopeptides were performed in female Wistar rats (6–8 weeks, 200–250 g). Approximately 1 MBq of each radiolabeled compound was administered per animal via intravenous injection. At designated time points, the animals were euthanized, and selected tissues (heart, lung, spleen, liver, kidney, muscle, intestine, brain, and stomach), bone, and blood were collected for radioactivity measurement using a γ-counter. The results were expressed as the percentage of injected dose per gram of tissue or blood [%ID/g].

## Results

**Fig. 1:**
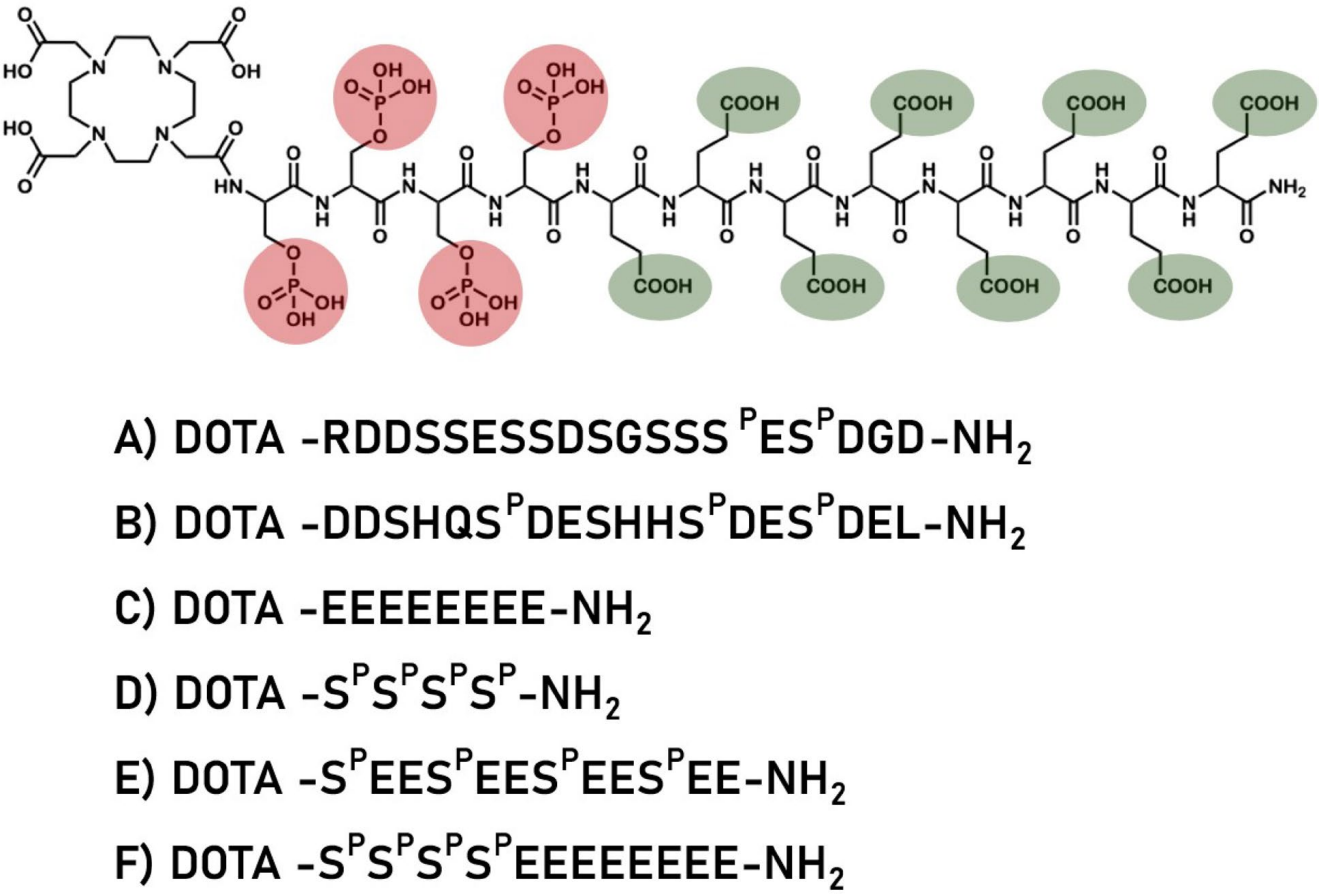
Chemical structure of DOTA-pS_4_E_8_ - the most effective peptide bone targeting peptide and the peptide sequences of (**A**) DOTA-MEPE-pASARM, (**B**) DOTA-OPN-pASARM, (**C**) DOTA-E_8_, (**D**) DOTA-pS_4_, (**E**) DOTA-p(SE_2_)_4_ and (**F**) DOTA-pS_4_E_8_

For the development of new peptide-based radiopharmaceuticals, several peptides varying in peptide sequence, peptide length, negatively charged groups, bifunctional chelating agents, and phosphorylated amino acids (Fig. 1). Subsequently, the pharmacokinetics of the potential tracers were studied in rats and mice.

Radiolabeled bisphosphonates have been developed for molecular imaging and therapy of bone metastasis. Up to 50% of the administered dose was found to accumulate in bone tissue. The remainder was eliminated via the urine within 24 h.

### Effect of the Absence of Phosphorylation of MEPE-ASARM on Bone Hydroxyapatite-Binding in vivo

**Fig. 2:**
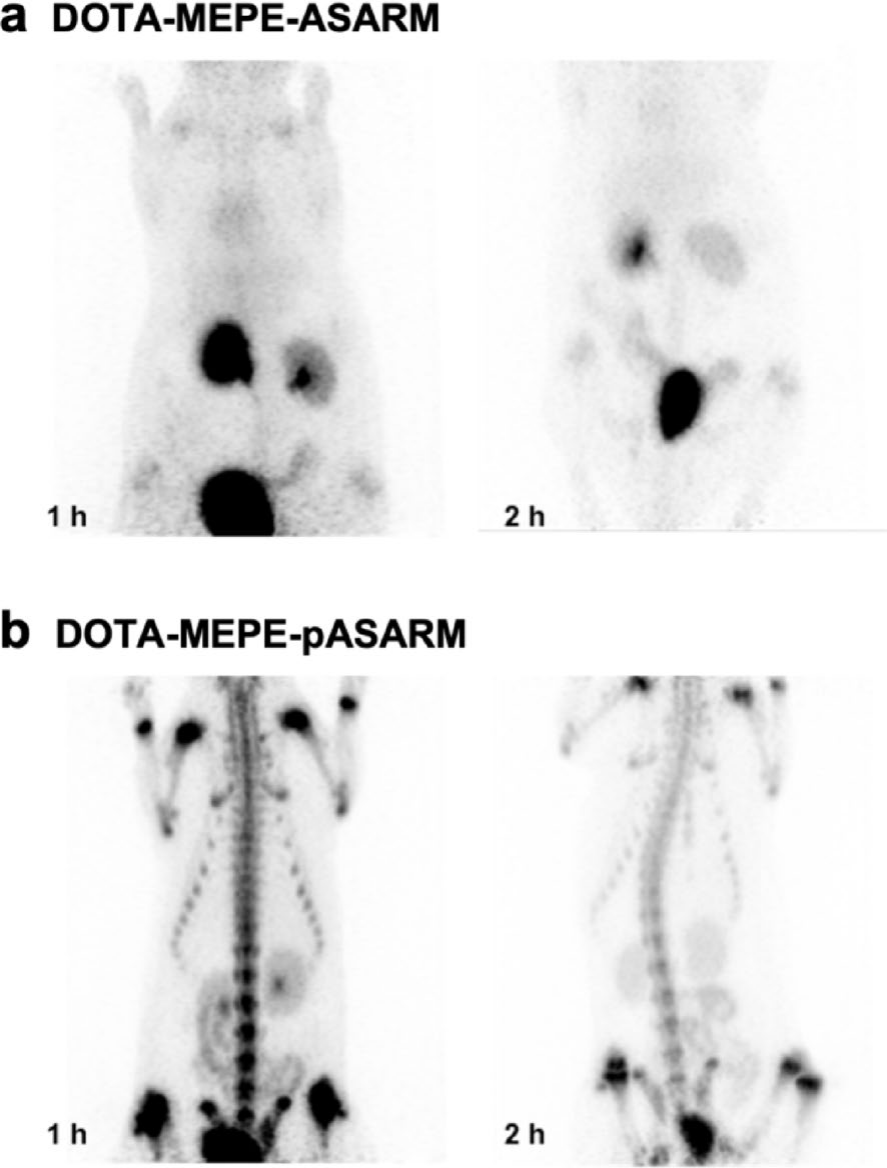
PET-images of the ^68^Ga-labeled non-phosphorylated MEPE-derived DOTA-ASARM conjugate (**a**) and its phosphorylated derivative (**b**) at 1 h and 2 h post-injection into female Wistar rats

Several authors have demonstrated the importance of post-translational modifications for the function of the SIBLING proteins in mineral-binding and crystal growth-regulating properties [28]. To study the effect of non-phosphorylated MEPE-ASARM peptide on hydroxyapatite crystal-binding in vivo, biodistribution studies of [^177^Lu]Lu-DOTA-YRDDSSESSDSGSSSESDGD ([^177^Lu]Lu-DOTA-ASARM) were performed in Wistar rats (Fig. 2).

At one and two hours post-injection, radioactivity was washed out via the kidneys with almost no signal in the bones.

### Bone hydroxyapatite-binding of phosphorylated opn-asarm peptide in vivo

**Fig. 3:**
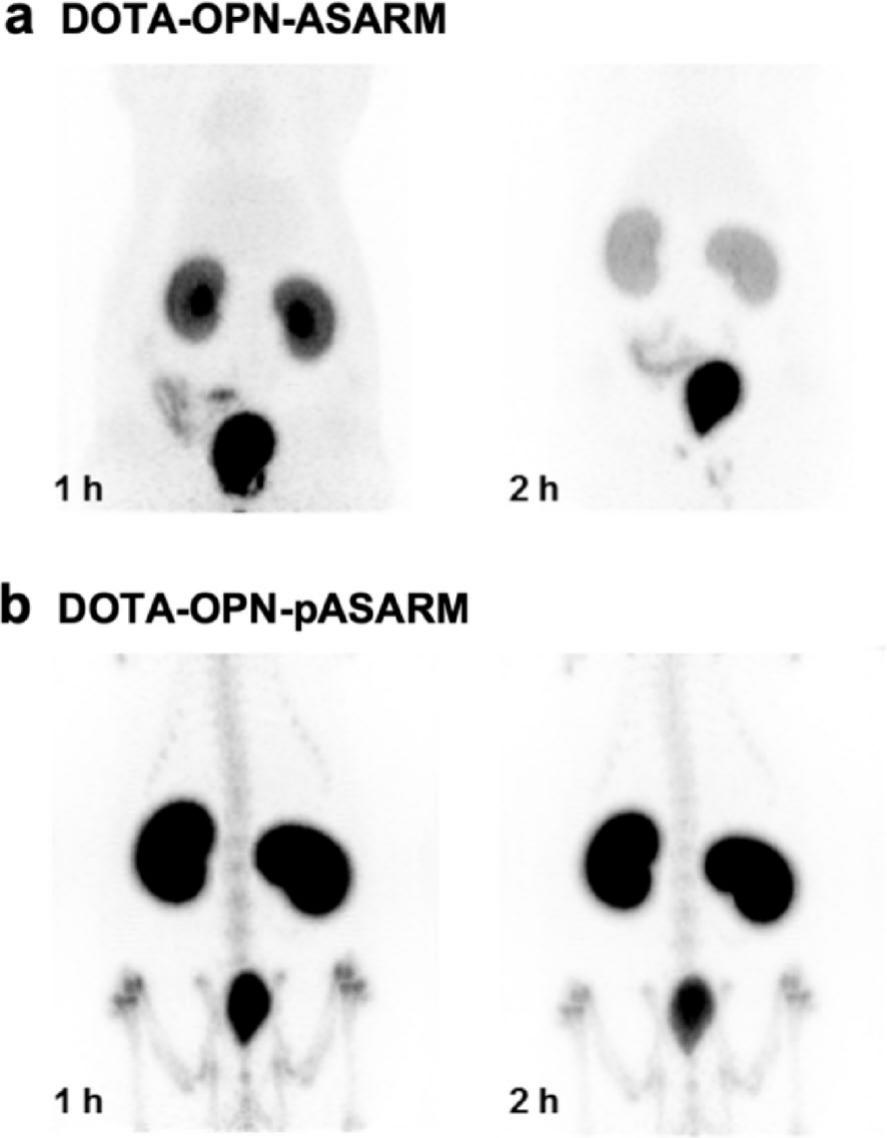
PET-images of the ^68^Ga-labeled non-phosphorylated (**a**) and phosphorylated (**b**) OPN-derived-DOTA-ASARM conjugate at 1 h and 2 h post-injection into female Wistar rats

In osteoblast culture mineralization, phosphorylated OPN-ASARM peptides have been identified as potent inhibitors of crystal growth and matrix mineralization [29]. We examined the bone mineral-binding effects of synthetic, triphosphorylated peptides based on the SIBLING protein OPN and their distribution using a small-animal PET (Fig. 2). This phosphorylated peptide OPN-ASARM (DDSHQpSDESHHpSDEpSDEL) shares 60% homology with the phosphorylated peptide MEPE-ASARM (RDDSSESSDSGpSSpSEpSDGD) and both contain three phosphoserine residues, five aspartic acid residues, and two/three glutamic acid residues. OPN-pASARM is characterized by a higher renal excretion and faster blood clearance than MEPE-pASARM. As described above, phosphorylation is required for bone hydroxyapatite-binding. The accumulation of phosphorylated [^68^Ga]Ga-DOTA-OPN-ASARM in the bone is higher at 1 and 2 h compared to the non-phosphorylated OPN-ASARM conjugate. Still, the triphosphorylated OPN-ASARM-derived conjugate showed lower bone uptake when compared to the triphosphorylated MEPE-ASARM-derived conjugate, indicating that the bone hydroxyapatite-binding potency is not only dependent on net negative charge but also on other peptide characteristics. In the phosphorylated OPN-ASARM peptide, an important structural feature is the presence of acidic amino acid serine residues-repeats [DSSESSDS]. The DSS and ESS repeats were identified as specific bone-targeting sequences in vitro [30]. The kidney uptake was high as described in Fig. 3, where the major fraction of the radioactivity of both DOTA-OPN-ASARM peptides (phosphorylated and non-phosphorylated) was detectable in the kidneys and the urinary bladder.

### Peptides of aspartic acid, glutamic acid, and DSS repeats

**Fig. 4:**
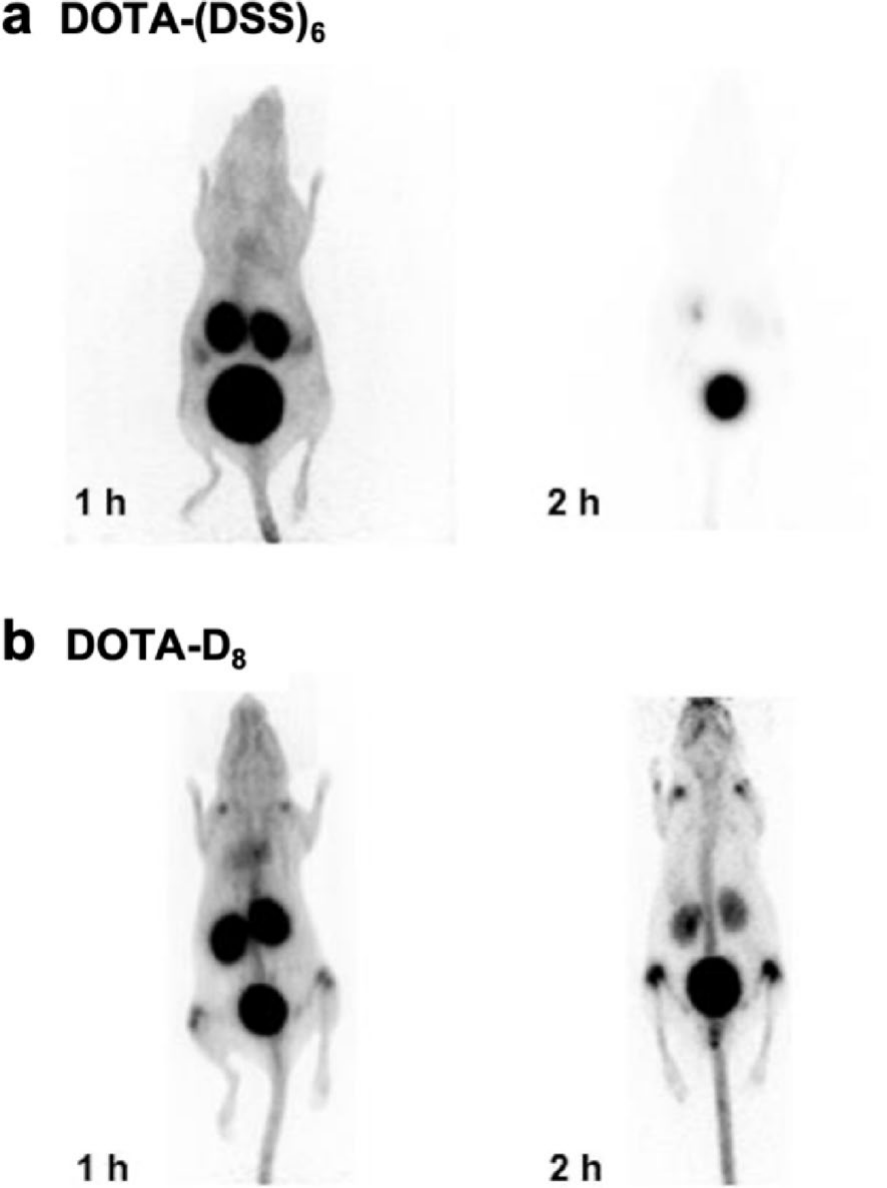
PET-images of [^68^Ga]Ga-DOTA-(DSS)_6_ (**a**), [^68^Ga]Ga-DOTA -D_8_ conjugate (**b**) at 1 h and 2 h post-injection into female NMRI mice

There are conserved peptides that have been identified as functional domains responsible for calcium regulation during the mineralization process, e.g., peptides containing aspartic acid, glutamic acid, or aspartic acid-serine-serine repeats. The proteins with extensive stretches of aspartic acid, glutamic acid, or aspartic acid-serine-serine repeats contribute to mineralization processes in vivo through their negative charge. Similarly to bisphosphonates, due to their affinity to the bone mineral, the peptides of aspartic acid or aspartic acid-serine-serine repeats have been conjugated to therapeutics via the peptide’s alpha-amino group for the bone delivery of the drug [31–34]. Recent studies in the hydroxyapatite binding assay have shown the specific bone-like mineral-binding of the peptides of DSS/ESS repeats in comparison to similar repeated sequences such as NTT, DTT, ETT, NSS, DAA, ASS, and NAA, and the affinity increases with the number of repeats [30]. To explore the potential mineral-binding ability of these peptides in vivo environments, we performed biodistribution studies of [^68^Ga]Ga-DOTA-(DSS)_6_ and [^68^Ga]Ga-DOTA-D_8_ in NMRI mice (Fig. 4a and b) and [^68^Ga]Ga-DOTA-E_8_ in Wistar rats (Supplemental Fig. 1). In contrast to non-phosphorylated ASARM peptides and repeating sequences of DSS, the unmodified conjugate of [^68^Ga]Ga-DOTA-D8 and [^68^Ga]Ga-DOTA-E8 showed some radioactivity in bone.

### Phosphopeptides of aspartic acid, glutamic acid, and DSS repeats

**Fig. 5:**
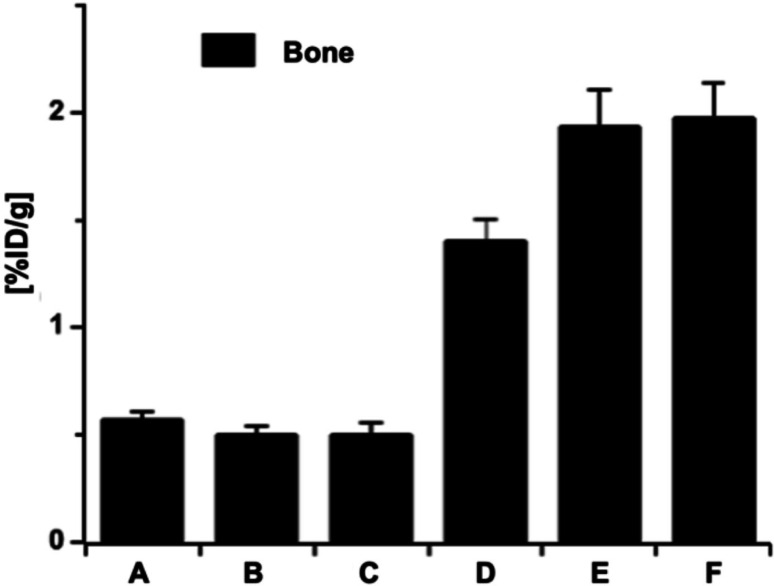
Accumulation of different ^177^Lu-labeled phosphorylated peptides of DOTA-MEPE-pASARM (**A**), DOTA-pS_2_(ESS)_4_ (**B**), DOTA-pS_4_D_8_ (**C**), DOTA-pS_2_E_8_ (**D**), DOTA-pS_4_E_8_ (**E**), and [^99m^Tc]Tc-MBP as reference (**F**) in the bone at 1 h post-injection into female Wistar rats

As noted in vivo before, the phosphorylation of the ASARM peptide is required for its affinity to hydroxyapatite. Other aspartic acid or glutamic acid oligomers have also been shown to have some affinity to hydroxyapatite. One study has demonstrated that a domain of at least eight glutamic acids is required for the nucleation of hydroxyapatite [35]. We investigated the phosphorylation-dependent mineral-binding potency of different bone-specific species, such as aspartic acid, glutamic acid, and DSS repeats. For the determination of the bone mineral-binding potency of phosphorylated peptides, the model peptides DOTA-pS_4_D_8_, DOTA-pS_2_E_8_, DOTA-pS_4_E_8,_ and DOTA-pS_2_(DSS)_4_ were synthesized, and in vivo biodistribution studies of [^177^Lu]Lu-DOTA-MEPE-pASARM, [^177^Lu]Lu-DOTA-pS_4_D_8_, [^177^Lu]Lu-DOTA-pS_2_E_8_, [^177^Lu]Lu-DOTA-pS_4_E_8,_ and [^177^Lu]Lu-DOTA-pS_2_(ESS)_4_ were performed in Wistar rats (Fig. 5; Table 1). Additional data from the biodistribution studies are provided in Supplemental Table 1. This table also includes raw [¹⁸F]NaF biodistribution data acquired at 1 h and 4 h post-injection. [¹⁸F]NaF exhibits a comparable soft-tissue distribution profile with very low organ uptake, but shows substantially lower renal accumulation at both time points (1 h: 0.14 ± 0.02%ID/g; 4 h: 0.02 ± 0.00%ID/g) compared to DOTA-pS_4_E_8_ (1 h: 1.16 ± 0.13%ID/g; 4 h: 0.93 ± 0.15%ID/g). [¹⁸F]NaF bone uptake, however, substantially exceeds that of both [⁹⁹ᵐTc]Tc-MBP and [^177^Lu]Lu-DOTA-pS_4_E_8_ (more than twice as high), reflecting its rapid bone accumulation, minimal renal excretion, and strong skeletal retention.

**Table 1:** Biodistribution studies of [^177^Lu]Lu-DOTA-phosphopeptide conjugates and bone standards [^99m^Tc]Tc-MBP and [^18^F]NaF. Female Wistar rats (*n* = 3) were injected i.v. with the radiolabeled conjugates (approx. 1 MBq). The radioactivity was measured at 1 h p.i.

	Compound	Blood [%ID/g]	Bone [%ID/g]*	Bone/Blood	Approx. Molar Acticity [GBq/mmol]
**A**	[^177^Lu]Lu-DOTA -MEPE-pASARM	0.05 ± 0.01	0.57 ± 0.04	11.40 ± 2.42	0.1
**B**	[^177^Lu]Lu-DOTA-pS_2_(ESS)_4_	0.05 ± 0.01	0.50 ± 0.04	10.00 ± 2.15	0.1
**C**	[^177^Lu]Lu-DOTA-pS_4_D_8_	0.06 ± 0.02	0.50 ± 0.06	8.33 ± 2.95	0.1
**D**	[^177^Lu]Lu-DOTA-pS_2_E_8_	0.03 ± 0.00	1.41 ± 0.10	47.00 ± 3.33	0.1
**E**	[^177^Lu]Lu-DOTA -pS_4_E_8_	0.03 ± 0.00	1.94 ± 0.17	64.67 ± 5.67	0.1
**F**	[^99m^Tc]Tc-MBP	0.11 ± 0.01	1.98 ± 0.16	18.00 ± 2.19	7
	[^18^ F]NaF	0.06 ± 0.01	4.54 ± 1.07	151.33 ± 61.78	50

* Knee region values were selected as representative of overall bone accumulation

As evident in Fig. 5 and Table 1, phosphate groups increase the peptides’ affinity for hydroxyapatite. In comparison to [^177^Lu]Lu-DOTA-MEPE-pASARM, the conjugates [^177^Lu]Lu-DOTA-pS_4_D_8_ and [^177^Lu]Lu-DOTA-pS_2_(ESS)_4_ showed similar bone mineral-binding potency. Notably, the bone-to-blood ratios (Table 1) highlight the favorable distribution characteristics of glutamate‑rich phosphopeptides: DOTA‑pS_4_E_8_ and DOTA‑pS_2_E_8_ showed the highest bone-to-blood ratios (64.67 ± 5.67 and 47.00 ± 3.33, respectively), reflecting rapid blood clearance combined with high and selective bone accumulation. In contrast, conjugates containing aspartic acid or ESS repeats showed lower ratios (8.33–11.40), indicating less efficient bone targeting and greater residual activity in circulation. While the standard [^99m^Tc]Tc-MBP showed high %ID/g values for bone biodistribution, its bone-to-blood ratio was comparatively low (18.00 ± 2.19), indicating longer circulation and higher residual activity. [¹⁸F]NaF exhibited exceptionally high bone%ID/g (4.54 ± 1.07) and bone-to-blood ratios (151.33 ± 61.78), serving as a clinical benchmark for ultra-fast clearance. The substitution of the ESS repeats with the anionic glutamic acid repeats significantly increased mineral-binding affinity, although replacement of this domain with the anionic aspartic acid repeats did not alter bone mineral-binding affinity, indicating a specific binding of the glutamic acid repeats to bone hydroxyapatite.

To further contextualize these findings against bisphosphonate-based therapeutic conjugates, we compared our knee bone uptake values to cited data for [^177^Lu]Lu-DOTA-zoledronate ([^177^Lu]Lu-DOTA^ZOL^) in Wistar rats at 1 h p.i [36]. [^177^Lu]Lu-DOTA^ZOL^ exhibited superior absolute femur uptake of 3.43 ± 0.41%ID/g (blood: 0.07 ± 0.01%ID/g; bone/blood 49.00 ± 9.13) compared to [^177^Lu]Lu-DOTA-pS_4_E_8_ femur shaft (0.67 ± 0.05%ID/g), although DOTA-pS_4_E_8_ knee uptake reached 1.94 ± 0.17%ID/g (bone/blood 64.67 ± 5.67). Rodent knee regions contain more cancellous bone than femoral shafts [37, 38]. Since the DOTA^ZOL^ study measured whole femurs (including cancellous-rich distal femur metaphysis and neck), our DOTA-pS_4_E_8_ knee region data provide a more direct comparison to their femur values than our femur shaft measurements [36].

Besides serine, tyrosine can be subjected to phosphorylation [39]. The novel phosphorylated conjugate [^68^Ga]Ga-DOTA-pY_4_E_8_ was synthesized to identify the ideal phosphorylated residues. The result of PET imaging, however, indicated that the phosphopeptide DOTA-pY_4_E_8_ exhibited a very low accumulation in the bone (Supplemental Fig. 2).

### Effect of glutamic and aspartic acid residues

To show the role of glutamic acid and aspartic acid residues in the phosphopeptides, DOTA-pS_2_ and DOTA-pS_4_ were synthesized and investigated in vivo. As illustrated in Supplemental Fig. 3, both phosphopeptides showed a loss of bone specificity.

### Effect of sulfation on hydroxyapatite binding

In addition to phosphorylation, other post-translational modifications, such as sulfation, glycosylation, and carboxylation, contribute to the increase of the net negative charge of the proteins as well. For instance, the sulfation of cysteic acid residues in bone sialoprotein is responsible for its high affinity to hydroxyapatite. To investigate the influence of sulfation in the peptides, an in vivo distribution study of the sulfated peptide [^177^Lu]Lu-DOTA-(Cya)_2_E_8_ was performed. Analysis of bone uptake shown in Supplemental Fig. 4 demonstrates that the sulfated peptide [^177^Lu]Lu-DOTA-(Cya)_2_E_8_) and [^177^Lu]Lu-DOTA-E8 exhibit minimal hydroxyapatite affinity, while [Lu]Lu-DOTA-pS_2_D_8_ displays substantially increased binding.

### Effect of phosphopeptide length

In osteoblast culture mineralization, Addison et al. demonstrated that the inhibitory effect of OPN-ASARM is dependent on ASARM phosphorylation, and this effect increases with the number of phosphoserine residues. The addition of phosphorylated groups further increases the total negative charge of the peptide and subsequently increases its affinity to hydroxyapatite. Based on these data, we examined how the length of the phosphopeptide modulates their properties, and how these changes may influence their pharmacokinetic profile.

In terms of potency and selectivity of phosphorylated peptide of glutamic acid repeats in mineral-binding in vivo, the bone uptake of the peptides [^177^Lu]Lu-DOTA-E_8_, [^177^Lu]Lu-DOTA-pSE_8_, [^177^Lu]Lu-DOTA-pS_2_E_8_, [^177^Lu]Lu-DOTA-pS_4_E_8_ and [^177^Lu]Lu-DOTA-pS_8_E_8_ were observed at 0.85 ± 0.10%ID/g, 1.41 ± 0.10%ID/g, 1.94 ± 0.17%ID/g and 1.37 ± 0.04%ID/g, respectively.

**Fig. 6:**
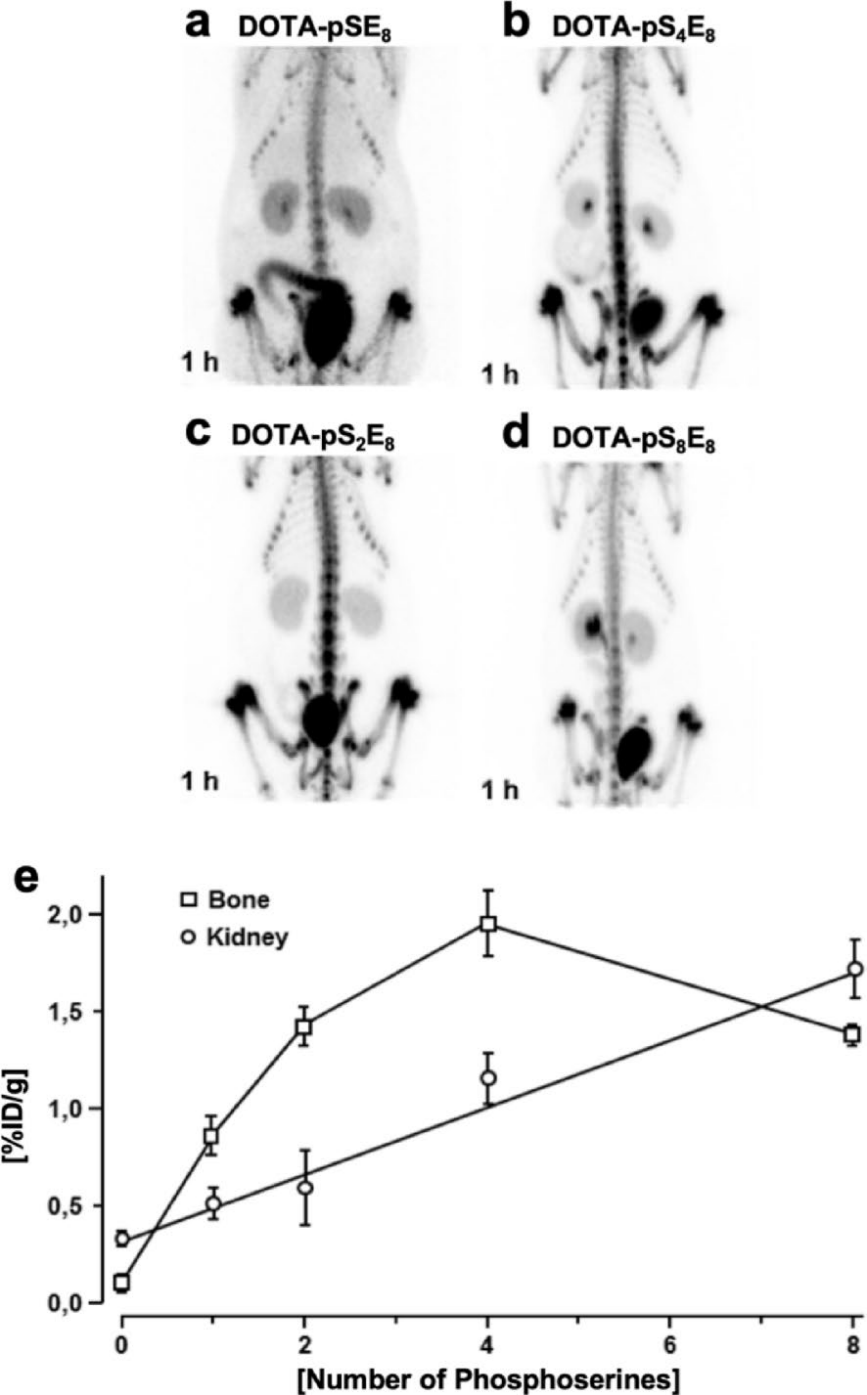
PET-images of the ^68^Ga-labeled phosphorylated conjugates with different sequence length DOTA-pS_n_E_8_ *n* = 1 (**a**), 2 (**b**), 4 (**c**), 8 (**d**) at 1 h post-injection into female Wistar rats. (**e**) Accumulation (%ID/g) of ^177^Lu-labeled phosphorylated peptides with different peptide lengths of DOTA-E_8_, DOTA-pSE_8_, DOTA-pS_2_E_8_, DOTA-pS_4_E_8,_ and DOTA-pS_8_E_8_ in bone (□) and in kidney (○) at 1 h post-injection into female Wistar rats

As described in Fig. 6e, besides the ability to adsorb on bone minerals, the kidney uptake increases with the length of phosphopeptide. In contrast to the correlation to the number of phosphorylated serine residues to mineral-binding, [^177^Lu]Lu-DOTA-pS_8_E_8_ showed a lower bone accumulation. These results were in line with the PET images of the ^68^Ga-labeled phosphorylated conjugates of different sequence lengths DOTA-pS_n_E_8_ (Fig. 6a-d).

### Phosphoserine position within glutamic acid repeats

The sites of phosphorylation are considered an important factor in the regulation of protein secretion and function. Many studies showed that most of the phosphorylated serine residues are highly conserved. However, the sites of the phosphoserine in ASARM peptides are distributed. To identify the ideal positions of the phosphorylated serine residues, the novel phosphorylated conjugate DOTA-(pSE_2_)_4_ was synthesized, where one phosphorylated serine residue was inserted on every second glutamic acid residue along the peptide sequence. After labeling with gallium-68, the distribution study in vivo was performed in Wistar rats. The results were compared to the previously synthesized related structures DOTA-pS_4_E_8_, which differ only in the phosphorylated serine residue positions. For the quantitative information, SUV values (maximum Standardized Uptake Values: SUV_max_) were determined 1 h after administration to evaluate the deposition of the compound in bone.

One hour post administration of DOTA-(pSE_2_)_4_, an enhanced signal was observed in the PET images of the kidneys and urinary bladder, while a reduced signal was observed in the bones when compared to DOTA-pS_4_E_8_ (Supplemental Fig. 5). The SUV_max_ in bone of [^68^Ga]Ga-DOTA-pS_4_E_8_ and [^68^Ga]Ga-DOTA-(pSE_2_)_4_ was 13.7 and 10.1, respectively (Supplemental Fig. 5). The SUV values demonstrated a significantly lower uptake of DOTA-(pSE_2_)_4_.

### Dynamic PET phosphopeptide study

**Fig. 7:**
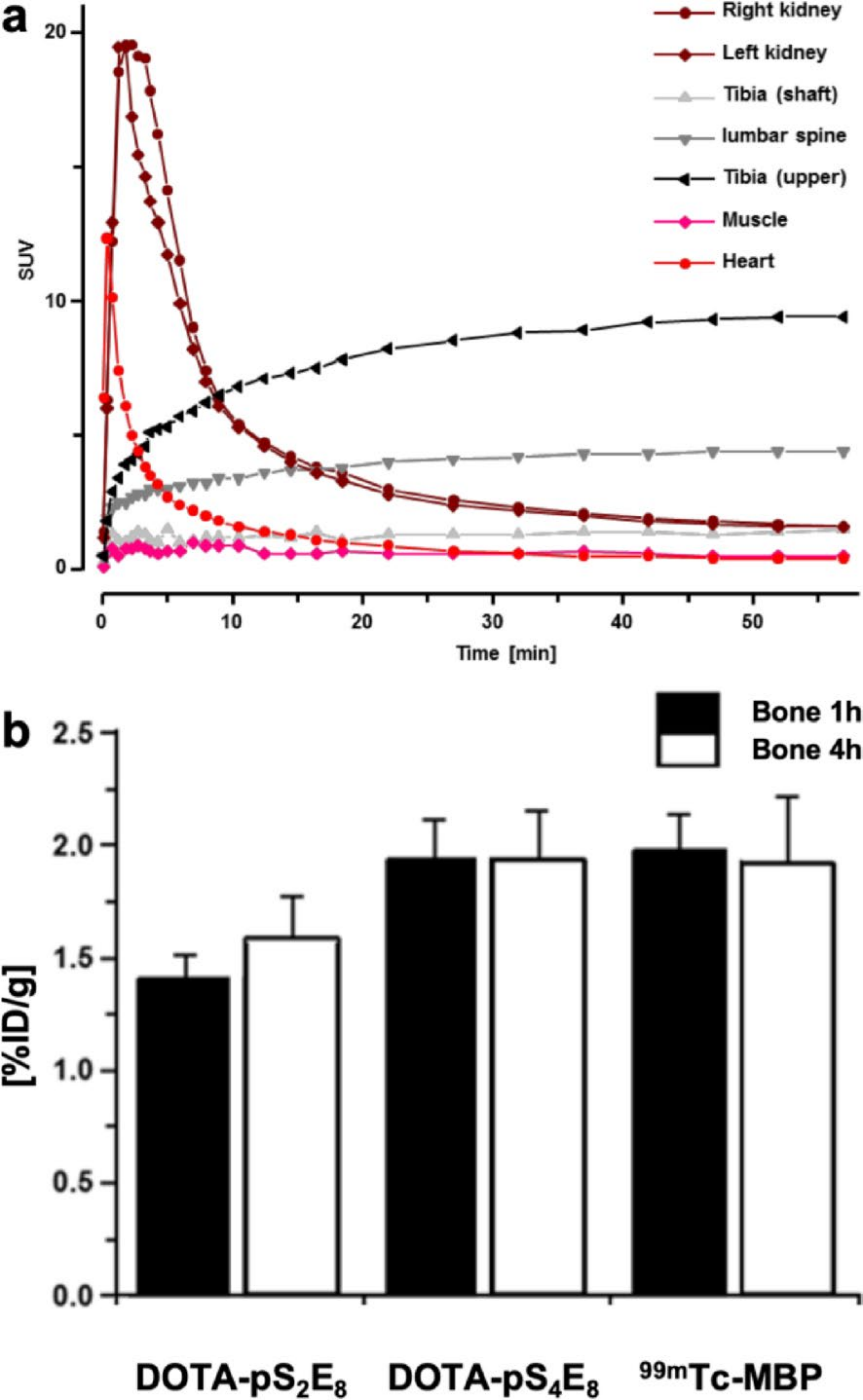
(**a**) Dynamic PET study of the ^68^Ga-labeled phosphorylated conjugate DOTA-pS_4_E_8_. (**b**) Accumulation (%ID/g) of the ^177^Lu-labeled phosphorylated peptides DOTA-pS_2_E_8_, DOTA-pS_4_E_8_ as compared to [^99m^Tc]Tc-MBP at 1 h and 4 h post-injection into female Wistar rats

As suggested by the results above, DOTA- pS_4_E_8_ exhibited the best bone-targeting properties. Therefore, to investigate this phosphopeptide further, animal micro PET scans of the phosphopeptide DOTA-pS_4_E_8_ were acquired dynamically over 55 min (Fig. 7a). PET imaging and biodistribution showed that the chemical phosphorylation of serines in the peptide sequence has a significant impact on the in vivo bone-targeting properties and pharmacokinetics. Similar to [^99m^Tc]Tc-MBP, [^68^Ga]Ga-/[^177^Lu]Lu-DOTA-pS_4_E_8_ showed specific accumulation in bone and no accumulation in muscle, lungs, liver, spleen, and intestine but lower non-specific tracer uptake in the kidney. In the biodistribution study, a stable accumulation in bone (Fig. 7b) could be demonstrated.

### Serum stability of phosphopeptides

The stability of the radiolabeled phosphopeptides was determined by incubation in fresh human serum. As shown in Supplemental Fig. 6, the radiophosphopeptides DOTA-pS_x_E_8_ (x = 1, 2 and 4) were completely stable in vitro for 24 h, whereas the phosphorylated DOTA-MEPE-pASARM showed signs of degradation.

## Discussion

In this study, we designed, synthesized, and evaluated a series of phosphopeptide based bone-targeting agents as an alternative to commonly used bisphosphonates. Our results demonstrate that the incorporation of phosphoserine moieties into peptide sequences, combined with the strategic clustering of negatively charged residues such as aspartic acid and glutamic acid repeats, significantly enhances their affinity for the bone mineral hydroxyapatite in vivo. Phosphorylation proved crucial for hydroxyapatite binding, as non-phosphorylated MEPE-ASARM and OPN-ASARM conjugates showed rapid renal clearance with negligible bone accumulation (Figs. 2, 3). Among all compounds tested, DOTA-pS_4_E_8_ exhibited the highest bone uptake at 1 h post-injection, reaching levels comparable to the clinically established gold standard methylene bisphosphonate ([^99m^Tc]Tc-MBP) (Figs. 5, 7). The comparative analyses focused on the 1 h post-injection time point because all data, especially the dynamic PET data (Fig. 7a), indicated that the bone uptake of the phosphopeptides plateaued between 1 and 2 h, whereas blood and soft-tissue signals decreased rapidly during the initial hour.

Non-phosphorylated ASARM peptides from both MEPE ([^177^Lu]Lu-DOTA-ASARM) and OPN ([^68^Ga]Ga-DOTA-OPN-ASARM) displayed predominant renal elimination within 1–2 h post-injection, with no detectable signal in the bone tissue (Figs. 2 and 3). While triphosphorylated MEPE-pASARM and OPN-pASARM both showed improved bone accumulation compared to their non-phosphorylated counterparts, MEPE-pASARM consistently outperformed OPN-pASARM, despite their 60% sequence homology and identical numbers of phosphoserine, aspartic acid, and glutamic acid residues (Figs. 2 and 3). These results indicate that bone accumulation of the MEPE-ASARM peptide depends on the presence of phosphorylated serine residues, which mediate specific interactions between phosphate groups and mineral hydroxyapatite. The difference between the performance of MEPE and OPN, however, suggests that hydroxyapatite binding potency depends not only on net negative charge but also on specific peptide characteristics, including the spatial arrangement of charged residues and structural motifs like the DSS/ESS repeats characteristic of OPN-derived sequences.

Unmodified aspartic/glutamic acid oligomers (DOTA-D_8_, DOTA-E_8_) showed modest bone accumulation, while DSS repeats (DOTA-(DSS)_6_) performed poorly in comparison (Fig. 4). Ogawa et al. previously demonstrated the bone-seeking potential of ^68^Ga-labeled oligo-aspartate/glutamate compounds in mice with high bone uptake and favorable blood-to-bone ratios [40]. Our biodistribution studies included DOTA-D_8_/E_8_ for comparison (Supplementary Table 1). Phosphopeptides substantially outperformed non-phosphorylated analogs, while avoiding their elevated kidney uptake, which Ogawa et al. targeted via glutamate stereoisomer modifications [41]. Phosphorylation of serine residues dramatically enhanced binding tendencies across all tested peptides. While DOTA-pS_4_D_8_ and DOTA-pS_2_(ESS)_4_ matched the binding potency of MEPE-pASARM, the replacement of DSS/ESS repeats with glutamic acid stretches (DOTA-pS_2_E_8_, DOTA-pS_4_E_8_) produced even higher hydroxyapatite affinity (Fig. 5). Consistent with these findings, the glutamate‑rich phosphopeptides displayed the highest bone-to-blood ratios (10.00–64.67), confirming their high bone selectivity and rapid blood clearance. A sequence of eight glutamic acid repeats appears to contribute substantially to enhanced hydroxyapatite affinity. Overall, these results demonstrate that, although all phosphopeptides share many negatively charged groups, structural differences among the peptides significantly influence bone binding. In addition to the phosphate groups, which are responsible for the general high affinity and selectivity of phosphopeptides for hydroxyapatite, these phosphopeptides also possess hydroxyl and carboxyl groups, which further enhance mineral binding by increasing local negative charge density and enabling additional coordination interactions.

Tyrosine phosphorylation in DOTA-pY_4_E_8_ resulted in very low bone accumulation (Supplemental Fig. 2), confirming the superiority of phosphoserine over other phosphorylated amino acids for hydroxyapatite binding. Simple phosphoserine oligomers (DOTA-pS_2_, DOTA-pS_4_) did not exhibit any bone specificity (Supplemental Fig. 3), demonstrating that extended acidic stretches are necessary to achieve bone affinity. Implementation of sulfates moieties in glutamic acid repeats in DOTA-(Cya)_2_E_8_ also failed to enhance binding compared with phosphorylated analogs like DOTA-pS_2_D_8_ (Supplemental Fig. 4). This demonstrates that negative charge modifications do not generally equivalently promote hydroxyapatite affinity. These results suggest that phosphorylation is highly suited for mineral coordination.

The [¹⁸F]NaF biodistribution data included in Table 1 and Supplementary Table 1 exemplify the exceptional performance of established ion-exchange-based bone imaging agents, achieving more than twofold higher bone accumulation than both MBP and our lead compound DOTA-pS_4_E_8_, alongside virtually complete renal clearance within 1 h p.i., as reflected in its exceptionally high bone-to-blood ratio (151.33 ± 61.78). This benchmark explains the existence of calcium-mimetic bone-seeking agents for PET imaging. However, this was beyond the scope of our peptide platform aimed at versatile bone-seeking carrier. Direct comparison with clinically validated bone-seeking agents such as [^177^Lu]Lu-DOTA^ZOL^ showed, regardless of whether femur shaft or knee region data is compared, the competitive bone targeting performance of DOTA-pS_4_E_8_, despite lower absolute uptake, with superior bone-to-blood selectivity (64.67 ± 5.67 vs. 49.00 ± 9.13) [36].

Peptide length optimization revealed a clear optimum at four phosphoserine residues within E_8_ stretches. Bone uptake increased with the number of phosphoserines from DOTA-E_8_ to DOTA-pS_4_E_8_, but then declined again for DOTA-pS_8_E_8_ (Fig. 6e). One explanation for the decline is likely increased renal excretion, as shown in Fig. 6e.

Moreover, optimizing the position of phosphoserine residues within the peptides further improved their binding performance. Clustered phosphorylation in DOTA-pS_4_E_8_ (SUVmax bone = 13.7) outperformed the distributed placement in DOTA-(pSE_2_)_4_ (SUVmax = 10.1), indicating that localized high-density phosphate arrays optimize the mineral binding geometry (Supplemental Fig. 5).

Dynamic PET imaging confirmed the potential of DOTA-pS_4_E_8_, showing rapid and stable bone accumulation that is comparable to [^99m^Tc]Tc-MBP, with minimal uptake in muscle, lungs, liver, spleen, or intestine, and low renal retention (Fig. 7). All phosphopeptides (DOTA-pS_x_E_8_, x = 1,2,4) demonstrated complete serum stability over 24 h, outperforming MEPE-pASARM which showed measurable degradation (Supplemental Fig. 6). The pharmacokinetic properties contrast favorably with the long biological half-lives of bisphosphonates.

Because of their similarities to solely Asp/Glu oligomers, oligoglutamic phosphopeptides are expected to have the same hydroxyapatite-binding mechanism via Ca²⁺ coordination, preferentially targeting resorption surfaces and high-crystallinity hydroxyapatite characteristics of osteolytic lesions [33, 40, 42]. This may overcome limitations of bisphosphonate-tracers like [^99m^Tc]Tc-MBP, which in some cases show false negatives in purely osteolytic metastases due to mainly accumulating in osteoblastic lesions [43]. In addition, acidic oligopeptides such as DOTA-pS_4_E_8_ possess several advantages over bisphosphonate-based vectors: their hydroxyapatite binding is strong but reversible, resulting in shorter and more controllable bone residence times (hours to days instead of years) that reduce long-term skeletal accumulation and are better suited for transient therapeutic targeting [44, 45]. Their affinity can be easily tuned by adjusting peptide length, as has been evident from Fig. 6, allowing fine tuning of pharmacokinetics and localization without altering the payload structure. Furthermore, acidic oligopeptides have been successfully applied clinically and preclinically for site-specific delivery of enzymes, drugs, and nanoparticle formulations to bone, confirming their versatility and efficacy in conditions such as osteolytic lesions, fractures, and bone metastases [46–48].

While this study was limited to healthy rodent models rather than bone metastasis or metabolic bone disease contexts, where pathological hydroxyapatite remodeling may alter binding dynamics, the performance of DOTA-pS_4_E_8_ nevertheless establishes a strong proof-of-concept for rationally designed phosphopeptides as next-generation bone-targeting vectors.

These findings position phosphopeptides as viable alternatives to bisphosphonates with strong hydroxyapatite affinity and customizable structure-property relationships. Further development should prioritize disease model validation, chelator optimization for therapeutic radionuclides (^177^Lu, ^90^Y), and advanced preclinical safety evaluation to determine their clinical value.

## Conclusion

This study established phosphopeptides as a promising and tunable class of bone-targeting agents that substantially expand the potential of previously leading peptide sequences such as aspartic/glutamic acid oligomers and ASARM for targeting bone tumors. Our data showed that effective hydroxyapatite binding requires phosphorylation and is strongly influenced by sequence architecture. Phosphoserine provides superior mineral affinity compared with alternative negatively charged modifications. Optimization of phosphate number and positioning led to the identification of a lead construct with high bone uptake and favorable pharmacokinetics. The lead peptide, DOTA-pS_4_E_8_, achieved skeletal accumulation comparable to a clinically used bisphosphonate (MBP) and approaches the performance of [^177^Lu]Lu-DOTA^ZOL^, while displaying rapid clearance and minimal off-target retention. Their characteristics distinguish phosphopeptides from conventional bone-seeking agents, including Asp or Glu oligomers and ASARM peptides. They place phosphopeptides in the same league as bisphosphonates, with potential safety benefits due to expected biodegradability that require further investigation beyond the pharmacokinetics presented here [49]. These agents are promising candidates for further development in targeted delivery of diagnostic and therapeutic radionuclides for metastatic bone disease and other bone-related pathologies, particularly in cases where conventional bisphosphonate-based vectors show limited efficacy, poor pharmacokinetics, or insufficient selectivity for specific bone microenvironments.

## Electronic Supplementary Material

Supplementary Material 1

## Data Availability

All data generated or analysed during this study are included in this published article and its supplementary information files.
