# Supplementary material for: Beyond bisphosphonates: radiolabeled phosphopeptides for bone targeting
Source: EJNMMI Res. 2026 Apr 26;16:69. doi: 10.1186/s13550-026-01437-5 (PMC13111735; doi:10.1186/s13550-026-01437-5)
Supplement: Supplementary file 1 — Supplementary Material 1 [file 13550_2026_1437_MOESM1_ESM.docx]

Supplementary Information

****Beyond Bisphosphonates: Radiolabeled Phosphopeptides for Bone Targeting****

Mazen Jamous^1^*, Barbara Roether^1^*, Eric Mühlberg^2^, Christian Kleist^1^, Armin Kübelbeck^2^, Uwe Haberkorn^1^ and Walter Mier^1^

^1^Department of Nuclear Medicine, Heidelberg University Hospital, Im Neuenheimer Feld 400, 69120 Heidelberg, Germany

^2^Department of Pharmaceutical Technology and Biopharmacy, Ruprecht Karls University of Heidelberg, Im Neuenheimer Feld 329, 69120 Heidelberg, Germany

*Contributed equally to this work

**Contact:** Walter Mier (walter.mier@med.uni-heidelberg.de, phone +49 6221 567720)

**Methods**

HPLC Conditions

Analytical and preparative columns:

**C1**: Column: Chromolith Performance RP-18e column (3 × 100 mm), Merck (Darmstadt, Germany).

**C2**: Column: Atlantis C18 column (0.46 × 150 mm) Waters (Eschborn, Germany).

**C3**: Column: Hypersil Gold C18 column (0.21 × 200 mm) Waters (Eschborn, Germany).

**C4**: Column: XBridge BEH130 Prep C18 column (19 × 150 mm) Waters (Eschborn, Germany).

**C5**: Column: Nucleosil C4 column (150 × 4.6 mm), Macherey-Nagel (Düren, Germany).

The components: **A** = 0.1% TFA in water, **B** = 0.1% TFA in acetonitrile, **C** = 0.05% TFA in water, **D** = 0.05% TFA in acetonitrile, **E** = potassium phosphate buffer (20 mM, pH 7) and **F** = methanol were used as the mobile phase. Analytical gradients:

**G1**: flow 2 mL/min, **A** in 5 min (0%-100%) of **B**, RT, λ= 214 nm.

**G2**: flow 2 mL/min, **A** in 5 min (0%-100%) of **B**, RT, γ-detector.

**G3**: flow 0.2 mL/min, **C** in 30 min (0%-100%) of **D**, 60 °C, λ= 214 nm.

**G4**: flow 20 mL/min, **A** in 15 min (0%-30%) of **B**, RT, λ= 214 nm.

**G5**: flow 1 mL/min, **E** in 10 min (0%-100%) of **F**, RT, γ-detector.

**G6**: flow 1 mL/min, **A** in 10 min (0%-100%) of **B**, RT, γ-detector.

Solid-Phase Peptide Synthesis

The peptides were automatically prepared by solid-phase synthesis on a synthesizer (Applied Biosystems 433 A, Carlsbad, CA, USA) using the Fmoc strategy on a TentaGel R RAM resin (low capacity: 0.18 mmol/g, Rapp Polymere, Tübingen, Germany) as described previously (18). All Fmoc-protected amino acids were obtained from Orpegen Peptide Chemicals GmbH (Heidelberg, Germany), HATU/HBTU, and the pseudoproline dipeptides Fmoc-Asp(O*t*Bu)-Ser(Ψ^Me,Me^pro)-OH and Fmoc-Glu(O*t*Bu)-Ser(Ψ^Me,Me^pro)-OH from Novabiochem (Läufelfingen, Switzerland). For the synthesis of branched peptide conjugates, lysine was incorporated using orthogonally protected lysine derivatives (Iris Biotech GmbH, Marktredwitz, Germany).

Amino Acid Coupling

For the manual coupling of amino acids, a solution of four equivalents of amino acid in DMF, which was preactivated for 2 min with 3.9 equivalents of HBTU and 4 equivalents of DIPEA was added to the resin-attached peptide. The coupling mixture was shaken at RT for 20 min. The resin was washed with DMF (3 × 1 ml), DCM (3 × 1 ml), and diethyl ether (3 × 1 ml), and, finally, it was dried under vacuum.

DOTA Coupling

DOTA was inserted using DOTA-tris(*t*Bu ester) or DOTA-PNP (16). The prochelator DOTA-tris(*t*Bu ester) was coupled using DIPEA as base and HATU as activating agent (17). A solution of two equivalents of the prochelator DOTA-tris(*t*Bu ester) in DMF, which was preactivated for 2 min with 1.9 equivalents of HATU and 20 equivalents of DIPEA was added to the resin-attached peptide. The coupling mixture was shaken at RT for 4 h. The resin was washed with DMF (3 × 1 ml), DCM (3 × 1 ml), and diethyl ether (3 × 1 ml), and, finally, it was dried under vacuum. The active DOTA ester was coupled using DIPEA as a base without an activating agent. A solution of two equivalents of DOTA-PNP in DMF and 10 equivalents of DIPEA was added to the resin-attached peptide, and the coupling mixture in the syringe was shaken at RT for 4 h. The resin was washed with DMF (3 × 1 ml), DCM (3 × 1 ml), and diethyl ether (3 × 1 ml), and, finally, it was dried under vacuum.

Deprotection and Cleavage of the Peptide

The maleimide-containing peptides were cleaved from the resin and deprotected by incubation with concentrated trifluoroacetic acid (TFA) (95%). A solution of TFA/TIS/H_2_O (95:2.5:2.5) was added to the dried resin. The reaction mixture was shaken at RT for 1 h (17,19). The peptide was precipitated cold using diethyl ether. For the first purification step, the peptide was resuspended and centrifuged at 4000 rpm at 4 °C for 5 min twice.

Radiolabeling of Peptides

Radiolabeling of the peptides was performed with lutetium-177 (half-life 6.71 d; β-emission intensity (79%) for therapy and γ-emission intensity (11% and 6.4%), mean energies are 113 and 208 keV, for SPECT imaging). [^177^Lu]LuCl_3_ in 0.05 M HCl was obtained from Perkin Elmer (Rodgau, Germany), gallium-68 (half-life 68 min; positron intensity, 89% for PET-molecular imaging; mean positron energy, 830 keV) was obtained as [^68^Ga]GaCl_3_ from a ^68^Ge/^68^Ga generator (IDB Holland, The Netherlands. The analytical reversed-phase HPLC analyses of all radiolabeled peptides were carried out on an Agilent 1100 series HPLC system using the HPLC conditions **C1** and **G2**.

Radiolabeling of Phosphopeptides and MBP with ^99m^Tc, ^68^Ga, and ^177^Lu

The ^68^Ga-labeled phosphopeptides were prepared by incubation of each peptide (5 µl, 5 mM in DMSO) with gallium-68 (1 ml, 400-800 MBq), which was eluted with 1 mL of HCl (0.6 N), for 5-20 min at 95 °C. The pH was adjusted to 3.6-4 with sodium acetate buffer (200 µl, 2.5 M). After quality control, the radiolabeling solution was evaporated to obtain a small volume and diluted with PBS for the following studies. The preparation of ^177^Lu-labeled phosphoconjugates was performed by dilution of each phosphopeptide (2 µl, 5 mM) in 25 µl of sodium acetate buffer (0.4 M, pH 5), followed by incubation with [^177^Lu]LuCl_3_ (1-40 MBq) for 25 min at 95 °C. [^99m^Tc]Tc-MBP was prepared from ROTOP-MDP kits by adding 2 GBq [^99m^Tc]NaTcO_4_ in 1 mL saline to 5 mg MDP (285 mM), followed by incubation for 30 minutes at room temperature. After quality control, the radiolabeled phosphopeptides, as well as MBP, were diluted with PBS and used without any purification step for the following studies.

**Results**


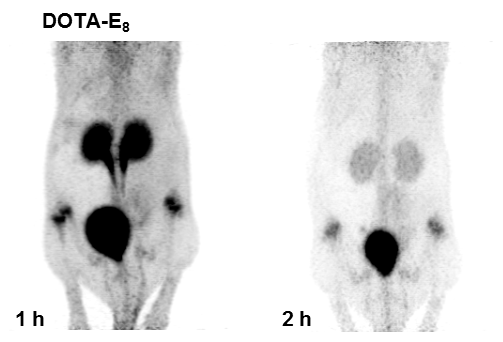


**Supplemental Figure 1.** PET images of the [^68^Ga]Ga-DOTA-E_8_ conjugate at 1 and 2 h post-injection into female Wistar rats.

The Effect of Different Types of Phosphorylated Amino Acids

The *O*-phosphorylation as a post-translational modification in the protein of the SIBLING family occurs primarily at the hydroxyl of serine, and only 2% of the phosphorylated residues are tyrosine, whereas the phosphorylation of other amino acid residues, including histidine, lysine, and glutamic acid, are chemically unstable (29). To identify the ideal phosphorylated residues, a novel phosphorylated conjugate, DOTA-pY_4_E_8,_ was synthesized. After labeling with gallium-68, the biodistribution study was performed in female Wistar rats. The obtained results (Supplemental Figure 2), including the qualitative PET images, indicate the loss of bone enrichment of the phosphopeptide DOTA-pY_4_E_8_.


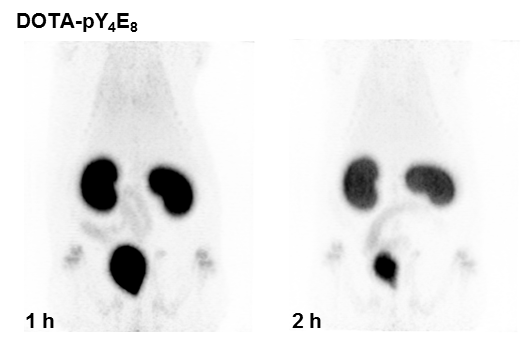


**Supplemental Figure 2.** PET images of the ^68^Ga-labeled phosphorylated DOTA-pY_4_E_8_ conjugate at 1 and 2 h post-injection into female Wistar rats.

.

**Supplemental Figure 3.** PET images of the ^68^Ga-labeled phosphorylated conjugates at 1 and 2 h post injection into female Wistar rats: (A) [^68^Ga]Ga-DOTA-pS_2_. (B) [^68^Ga]Ga-DOTA-pS_4_.

**Supplemental Table 1.** Biodistribution studies of [^177^Lu]Lu-DOTA-phosphopeptide conjugates and bone standards [^99m^Tc]Tc-MBP and [^18^F]NaF. Female Wistar rats (n = 3) were injected i.v. with the radiolabeled conjugates (approx. 1 MBq). The radioactivity was measured in bone and soft organs at 1 h and 4 h p.i.

|  | **Blood [%ID/g]** | **Heart [%ID/g]** | **Lung [%ID/g]** | **Spleen [%ID/g]** | **Liver [%ID/g]** | **Kidney [%ID/g]** | **Muscle [%ID/g]** | | **Intestine [%ID/g]** | **Brain [%ID/g]** | **Stomach [%ID/g]** | **Femur [%ID/g]** | **Knee [%ID/g]** |
| --- | --- | --- | --- | --- | --- | --- | --- | --- | --- | --- | --- | --- | --- |
| **[^18^F]NaF(1 h)** | 0.06 ±0.01 | 0.04 ±0.01 | 0.06 ±0.00 | 0.05 ±0.01 | 0.05 ±0.00 | 0.14 ±0.02 | 0.04 ±0.01 | 0.04 ±0.00 | | 0.01 ±0.00 | 0.05 ±0.02 | **2.52 ±0.90** | **4.54 ±1.07** |
| **[^18^F]NaF(4 h)** | 0.01 ±0.00 | 0.00 ±0.00 | 0.01 ±0.00 | 0.01 ±0.00 | 0.01 ±0.00 | 0.02 ±0.00 | 0.00 ±0.00 | 0.01 ±0.00 | | 0.01 ±0.00 | 0.06 ±0.05 | **2.40 ±0.20** | **4.80 ±0.27** |
| **[^177^Lu]Lu-DOTA-D_8_ (1 h)** | 0.11 ±0.07 | 0.02 ±0.00 | 0.06 ±0.02 | 0.03 ±0.01 | 0.03 ±0.00 | 0.28 ±0.03 | 0.01 ±0.00 | 0.04 ±0.01 | | 0.01 ±0.00 | 0.03 ±0.00 | **0.13 ±0.01** | **0.24 ±0.01** |
| **[^177^Lu]Lu-DOTA-E_8_ (1 h)** | 0.10 ±0.02 | 0.03 ±0.00 | 0.13 ±0.04 | 0.02 ±0.01 | 0.02 ±0.01 | 0.33 ±0.01 | 0.02 ±0.00 | 0.03 ±0.00 | | 0.00 ±0.00 | 0.03 ±0.02 | **0.03 ±0.01** | **0.08 ±0.03** |
| **[^177^Lu]Lu-DOTA-pASARM (1 h)** | 0.05 ±0.01 | 0.02 ±0.00 | 0.04 ±0.01 | 0.02 ±0.00 | 0.02 ±0.00 | 0.34 ±0.06 | 0.01 ±0.00 | 0.04 ±0.03 | | 0.00 ±0.00 | 0.04 ±0.05 | **0.17 ±0.03** | **0.57 ±0.04** |
| **[^177^Lu]Lu-DOTA-pASARM (4 h)** | 0.00 ±0.00 | 0.00 ±0.00 | 0.00 ±0.00 | 0.01 ±0.00 | 0.01 ±0.01 | 0.16 ±0.03 | 0.00 ±0.00 | 0.01 ±0.01 | | 0.00 ±0.00 | 0.00 ±0.00 | **0.13 ±0.03** | **0.46 ±0.09** |
| **[^177^Lu]Lu-DOTA-pS_1_E_8_ (1 h)** | 0.04 ±0.01 | 0.02 ±0.01 | 0.05 ±0.03 | 0.02 ±0.00 | 0.02 ±0.00 | 0.67 ±0.28 | 0.01 ±0.01 | 0.03 ±0.01 | | 0.00 ±0.00 | 0.05 ±0.04 | **0.28 ±0.05** | **0.85 ±0.10** |
| **[^177^Lu]Lu-DOTA-pS_2_E_8_ (1 h)** | 0.03 ±0.00 | 0.01 ±0.00 | 0.03 ±0.00 | 0.01 ±0.00 | 0.01 ±0.00 | 0.60 ±0.19 | 0.01 ±0.00 | 0.04 ±0.03 | | 0.00 ±0.00 | 0.05 ±0.04 | **0.47 ±0.02** | **1.41 ±0.10** |
| **[^177^Lu]Lu-DOTA-pS_2_E_8_ (4 h)** | 0.00 ±0.00 | 0.00 ±0.00 | 0.01 ±0.00 | 0.01 ±0.00 | 0.01 ±0.00 | 0.56 ±0.07 | 0.00 ±0.00 | 0.01 ±0.00 | | 0.00 ±0.00 | 0.00 ±0.00 | **0.52 ±0.10** | **1.59 ±0.18** |
| **[^177^Lu]Lu-DOTA-pS_4_E_8_ (1 h)** | 0.03 ±0.00 | 0.02 ±0.00 | 0.04 ±0.00 | 0.02 ±0.00 | 0.02 ±0.00 | 1.16 ±0.13 | 0.01 ±0.00 | 0.03 ±0.01 | | 0.00 ±0.00 | 0.05 ±0.05 | **0.67 ±0.05** | **1.94 ±0.17** |
| **[^177^Lu]Lu-DOTA-pS_4_E_8_ (4 h)** | 0.01 ±0.00 | 0.01 ±0.00 | 0.02 ±0.01 | 0.04 ±0.01 | 0.03 ±0.01 | 0.93 ±0.15 | 0.01 ±0.01 | 0.02 ±0.00 | | 0.00 ±0.00 | 0.01 ±0.01 | **0.65 ±0.13** | **1.94 ±0.21** |
| **[^177^Lu]Lu-DOTA-pS_8_E_8_ (1 h)** | 0.05 ±0.01 | 0.04 ±0.01 | 0.09 ±0.05 | 0.03 ±0.01 | 0.04 ±0.01 | 1.72 ±0.15 | 0.02 ±0.01 | 0.06 ±0.02 | | 0.01 ±0.01 | 0.12 ±0.08 | **0.52 ±0.06** | **1.37 ±0.04** |
| **[^177^Lu]Lu-DOTA-pS_4_D_8_ (1 h)** | 0.06 ±0.02 | 0.02 ±0.01 | 0.06 ±0.02 | 0.02 ±0.00 | 0.03 ±0.01 | 0.33 ±0.05 | 0.01 ±0.00 | 0.05 ±0.03 | | 0.00 ±0.00 | 0.07 ±0.07 | **0.15 ±0.02** | **0.50 ±0.06** |
| **[^177^Lu]Lu-DOTA-pS_2_(ESS)_4_ (1 h)** | 0.05 ±0.01 | 0.02 ±0.00 | 0.07 ±0.01 | 0.03 ±0.00 | 0.03 ±0.00 | 0.35 ±0.04 | 0.01 ±0.00 | 0.04 ±0.01 | | 0.00 ±0.00 | 0.12 ±0.12 | **0.23 ±0.04** | **0.50 ±0.04** |
| **[^99m^Tc]Tc -MBP (1 h)** | 0.11 ±0.01 | 0.03 ±0.00 | 0.10 ±0.00 | 0.17 ±0.04 | 0.12 ±0.02 | 0.80 ±0.17 | 0.02 ±0.00 | 0.06 ±0.02 | | 0.00 ±0.00 | 0.02 ±0.02 | **1.08 ±0.16** | **1.98 ±0.16** |
| **[^99m^Tc]Tc-MBP (4 h)** | 0.04 ±0.01 | 0.02 ±0.00 | 0.05 ±0.01 | 0.10 ±0.03 | 0.07 ±0.03 | 0.68 ±0.10 | 0.01 ±0.00 | 0.04 ±0.00 | | 0.00 ±0.00 | 0.05 ±0.04 | **0.94 ±0.06** | **1.92 ±0.30** |


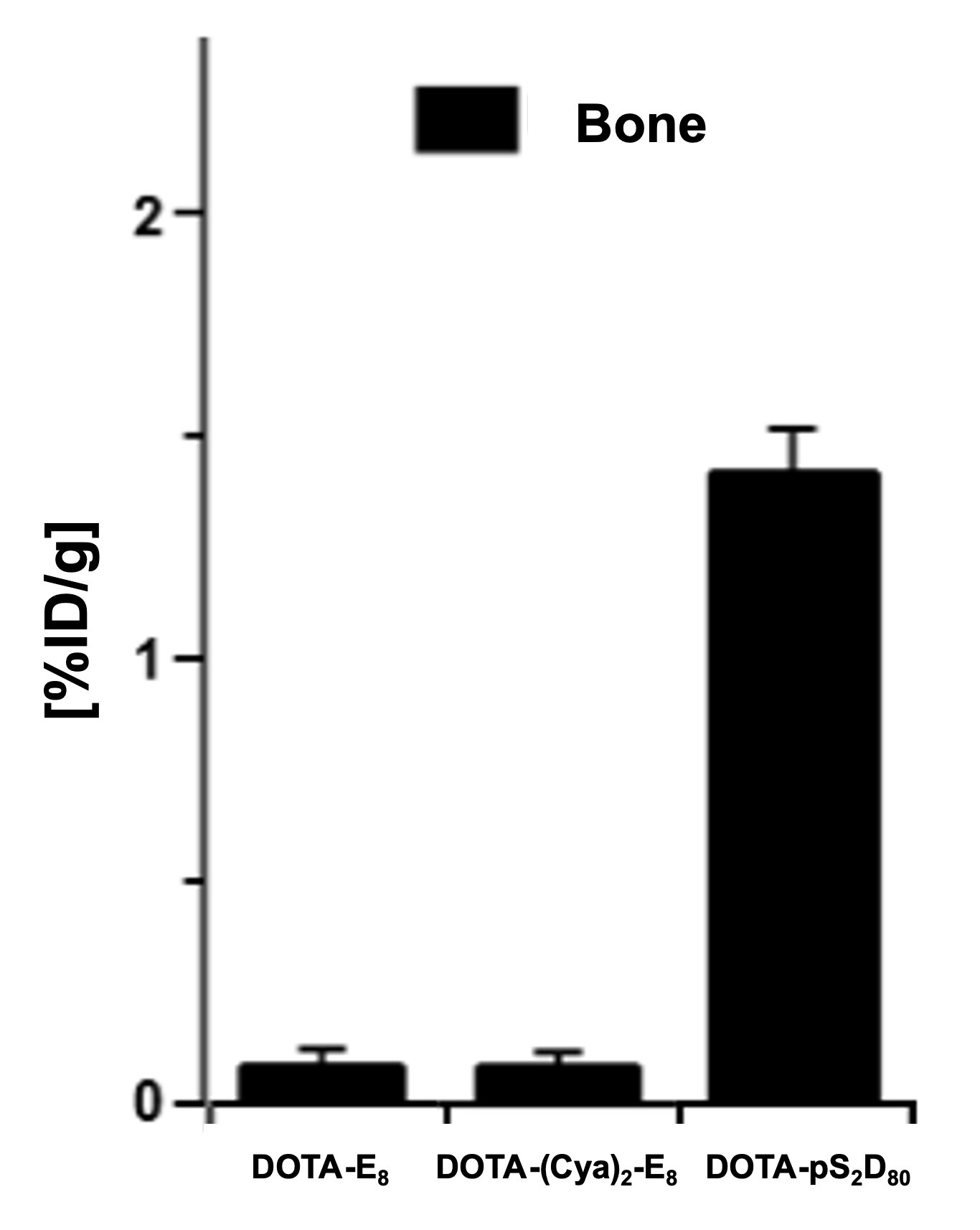


**Supplemental Figure 4.** The percentage accumulation (%ID/g) of different ^177^Lu-labeled peptides of DOTA-E_8_, DOTA-(Cya)_2_E_8_, DOTA-pS_2_D_8_ in the bone at 1 h post-injection into female Wistar rats.


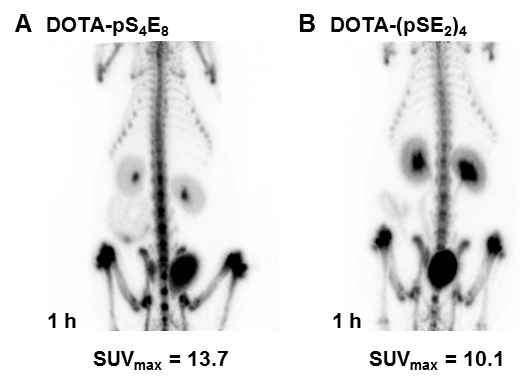


**Supplemental Figure 5.** PET images of the ^68^Ga-labeled phosphorylated conjugates with different sites of phosphorylation at 1 h post-injection into female Wistar rats: (A) DOTA-pS_4_E_8_. (B) DOTA-(pSE_2_)_4_.

Serum Stability of Phosphopeptides

The stability of the radiolabeled phosphopeptides was determined by incubation in fresh human serum at a concentration of 20 mmol/mL at 37 °C. Degradation of the radiolabeled peptides was assessed by radio-RP-HPLC. As shown in Supplemental Figure 6, the radiophosphopeptides DOTA-pS_x_E_8_ (x = 1, 2 and 4) were completely stable *in vitro* in human serum samples at 37 °C for 24 h. No new peaks or shoulders were observed by RP-HPLC analysis. In contrast, the analysis of the degradation pathway of the phosphorylated DOTA-MEPE-pASARM showed that this phosphopeptide underwent peptidase cleavage.

**Supplemental Figure 6.** Stability kinetics of phosphopeptide conjugates in human serum at 37 °C; (A) DOTA-pSE_8_; (B) DOTA-pS_2_E_8_; (C) DOTA-pS_4_E_8_**; (**D) DOTA-MEPE-pASARM. HPLC system conditions: (C5, G5).

**Compound Characterization**

Peptide conjugates were characterized by RP-HPLC and ESI/MS, confirming ≥ 90% purity for all compounds.


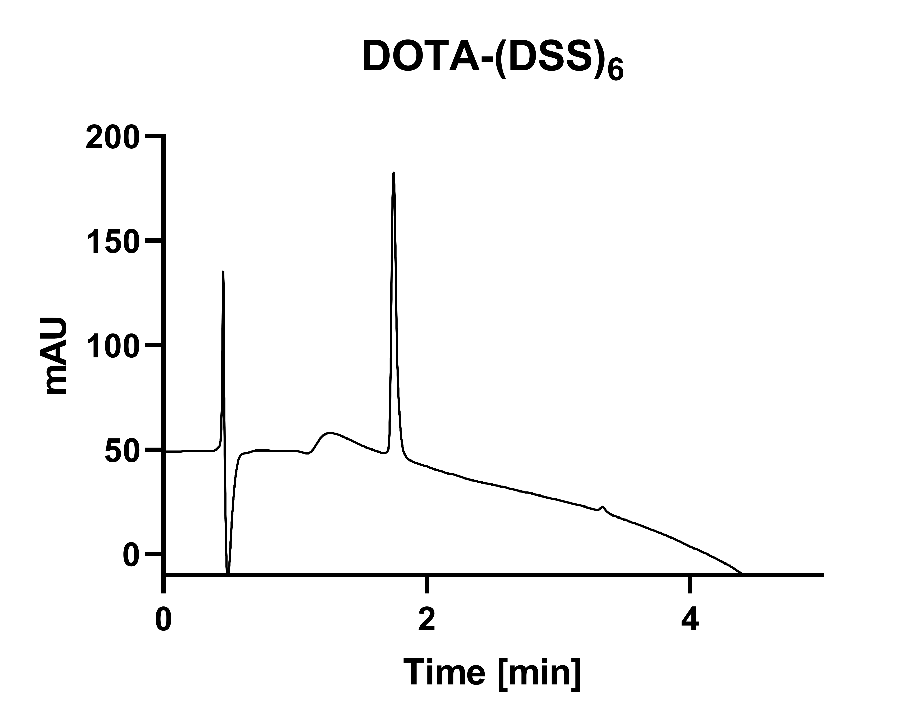


**Supplemental Figure 7.** RP-HPLC chromatogram of DOTA-(DSS)_6_ using conditions C1.


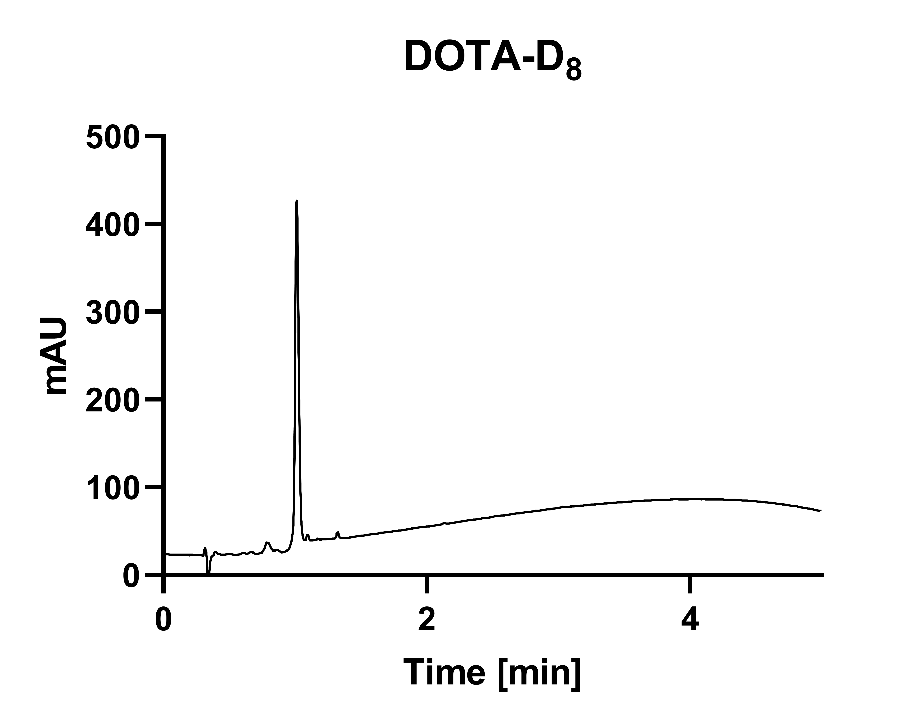


**Supplemental Figure 8.** RP-HPLC chromatogram of DOTA-D_8_ using conditions C1.
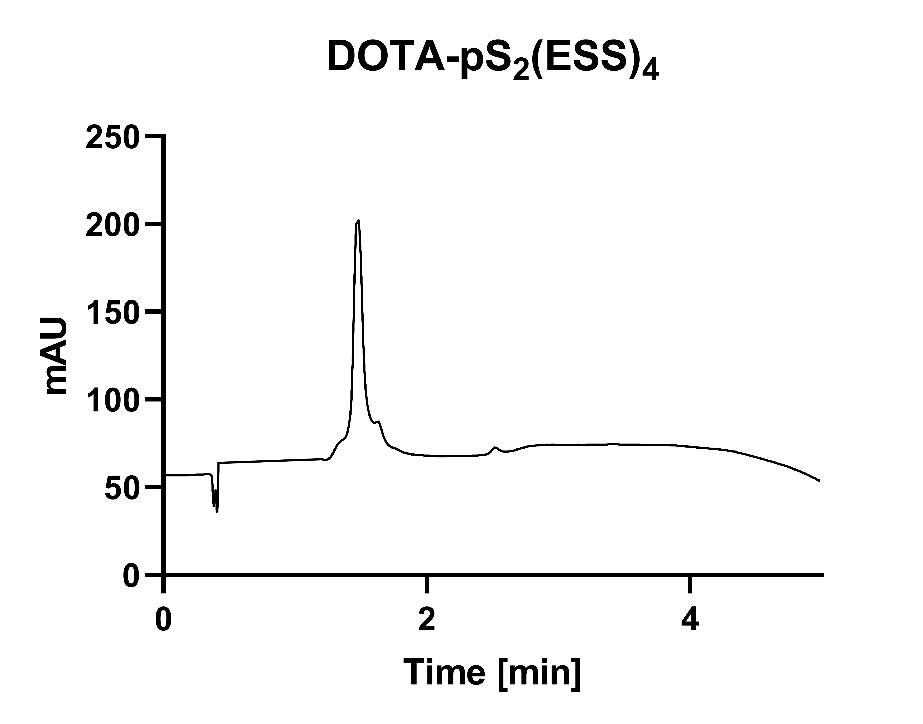


**Supplemental Figure 9.** RP-HPLC chromatogram of DOTA-pS_4_(ESS)_4_ using conditions C1.


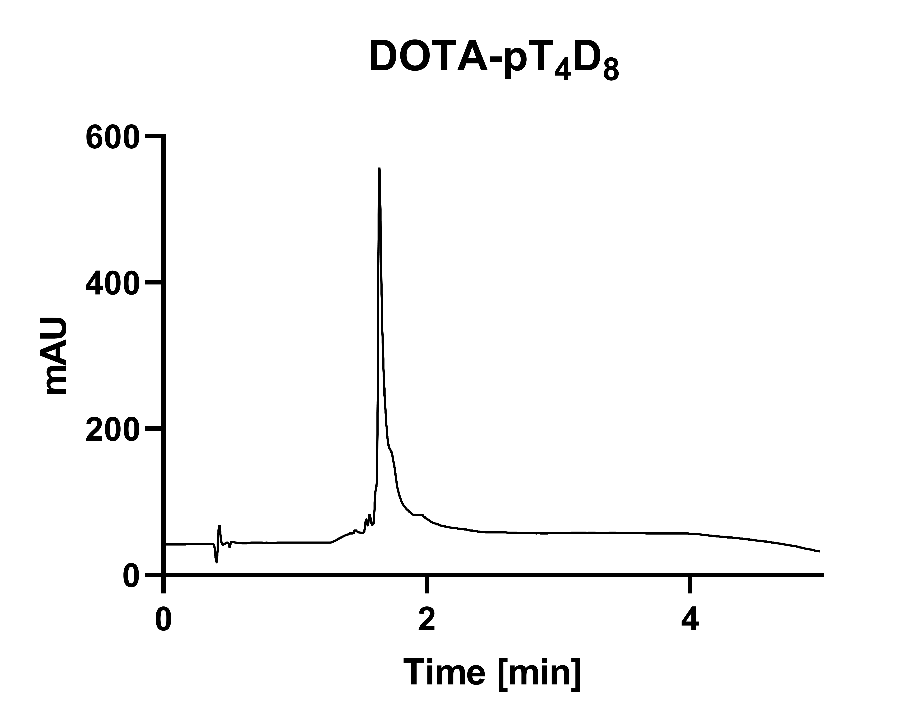

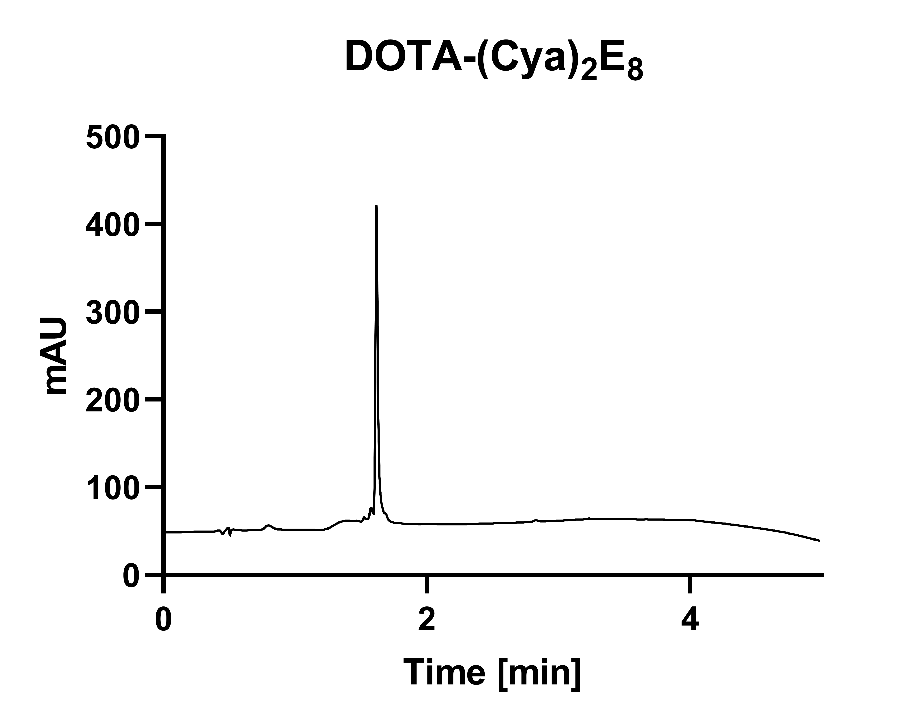
 **Supplemental Figure 10.** RP-HPLC chromatogram of DOTA-(Cya)_2_E_8_ using conditions C1.

**Supplemental Figure 11.** RP-HPLC chromatogram of DOTA-pT_4_D_8_ using conditions C1.


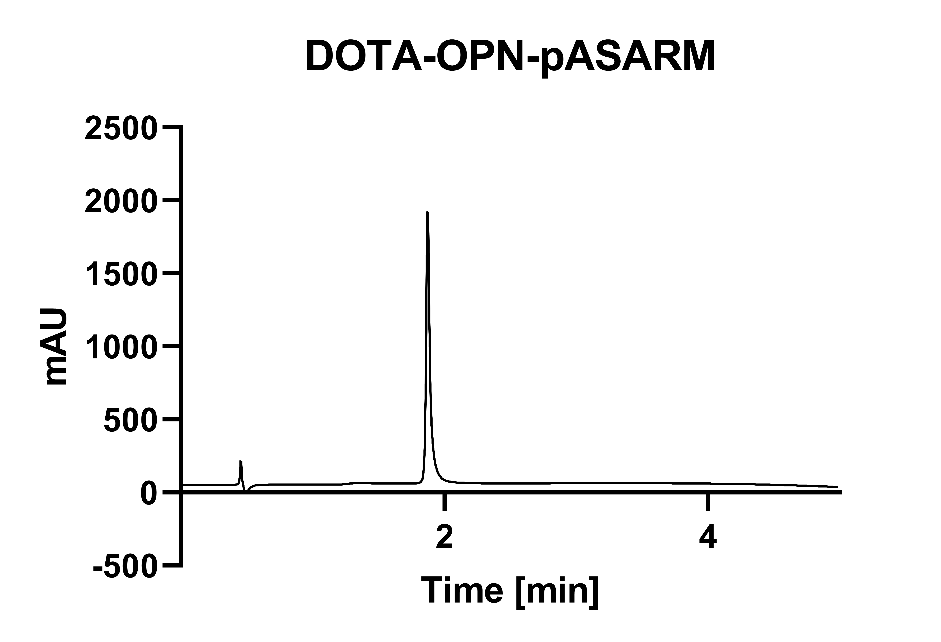

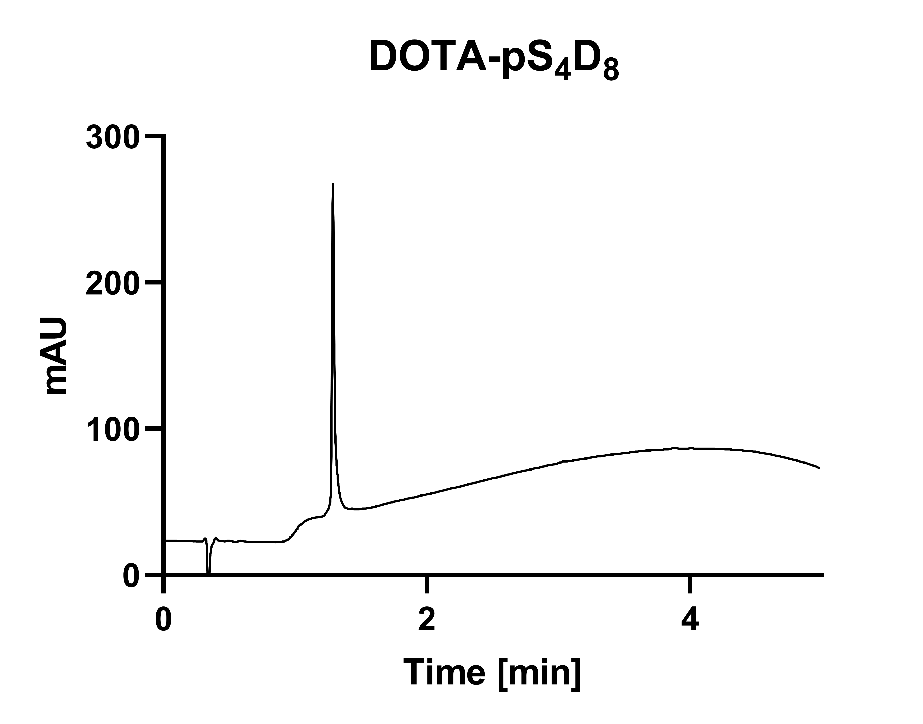
 **Supplemental Figure 12.** RP-HPLC chromatogram of DOTA-pS_4_D_8_ using conditions C1.

**Supplemental Figure 13.** RP-HPLC chromatogram of DOTA-OPN-pASARM using conditions C1.


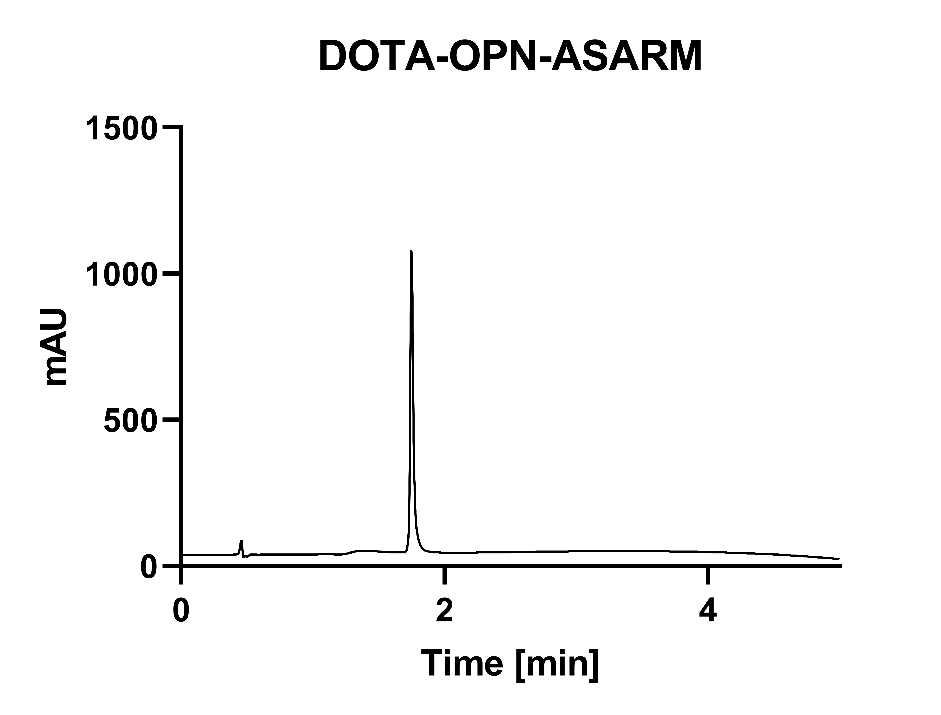

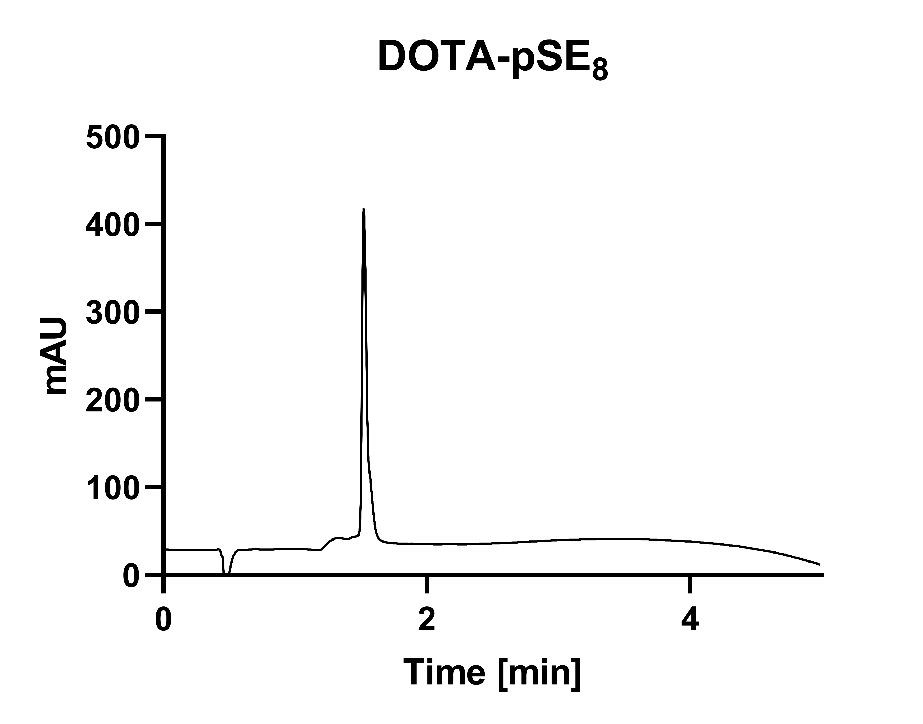
 **Supplemental Figure 14.** RP-HPLC chromatogram of DOTA-pSE_8_ using conditions C1.

**Supplemental Figure 15.** RP-HPLC chromatogram of DOTA-OPN-ASARM using conditions C1.


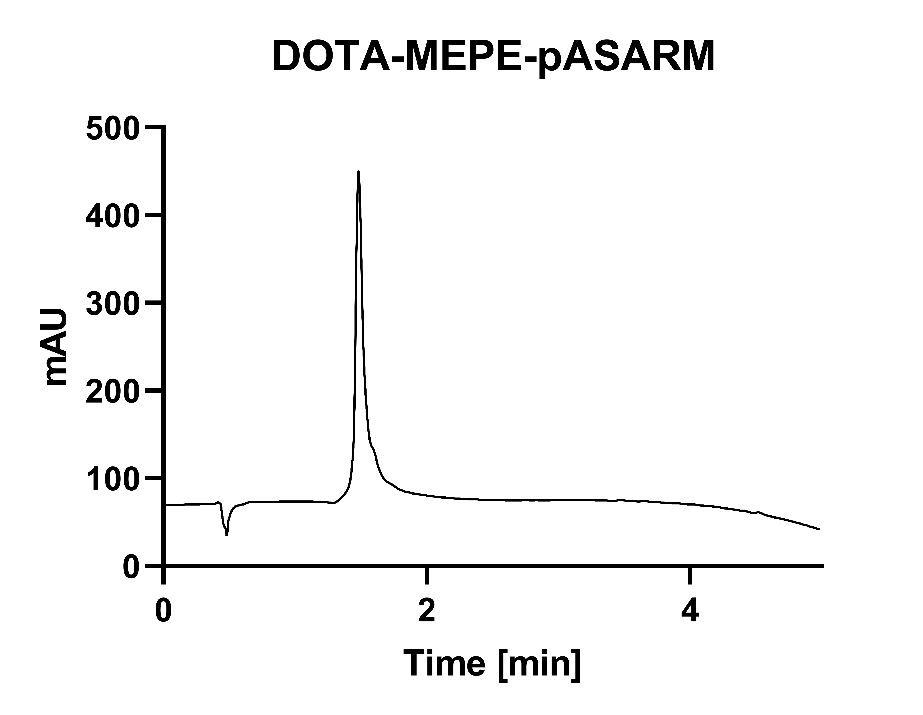

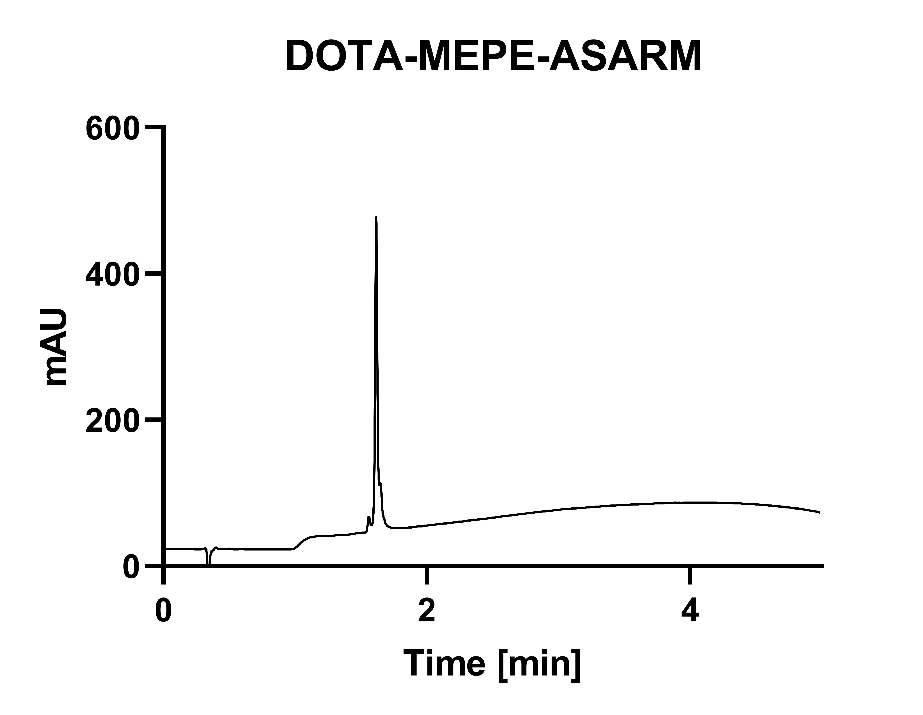
 **Supplemental Figure 16.** RP-HPLC chromatogram of DOTA-MEPE-ASARM using conditions C1.

**Supplemental Figure 17.** RP-HPLC chromatogram of DOTA-MEPE-pASARM using conditions C1.


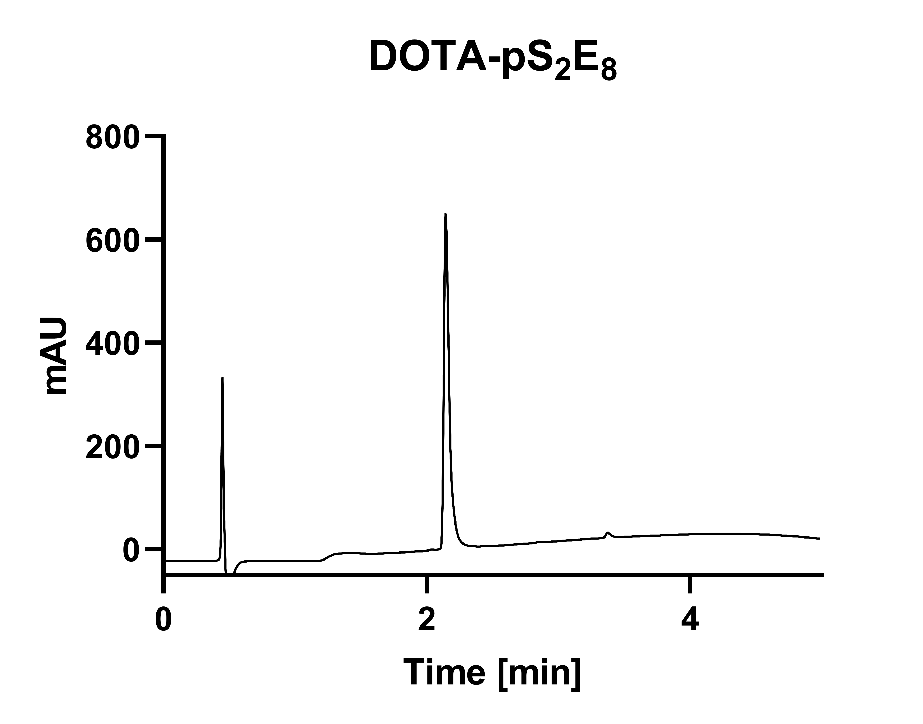

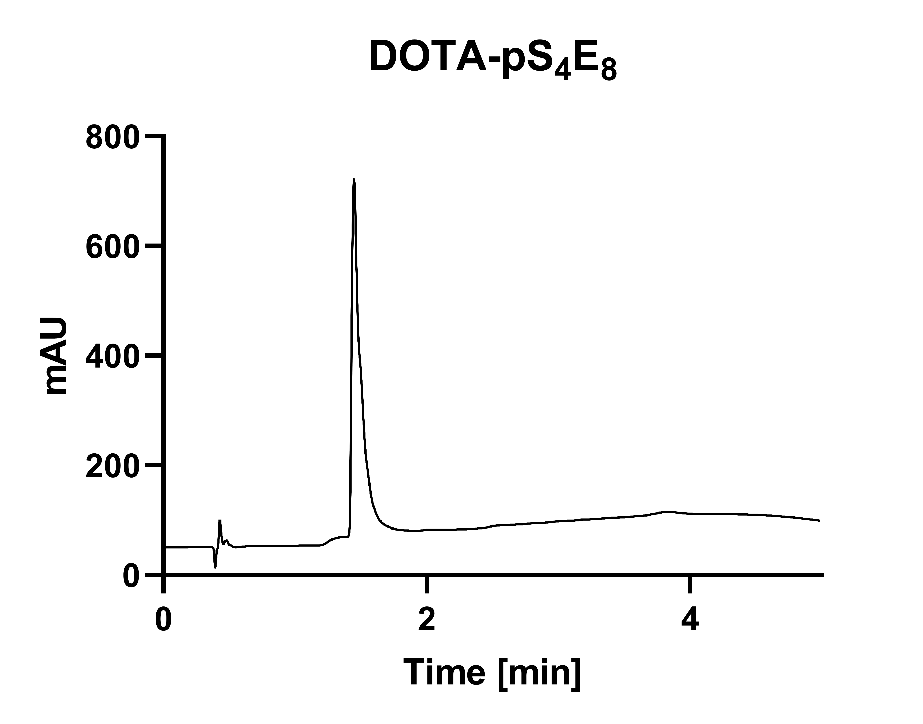
 **Supplemental Figure 18.** RP-HPLC chromatogram of DOTA-pS_4_E_8_ using conditions C1.

**Supplemental Figure 19.** RP-HPLC chromatogram of DOTA-pS_2_E_8_ using conditions C1.


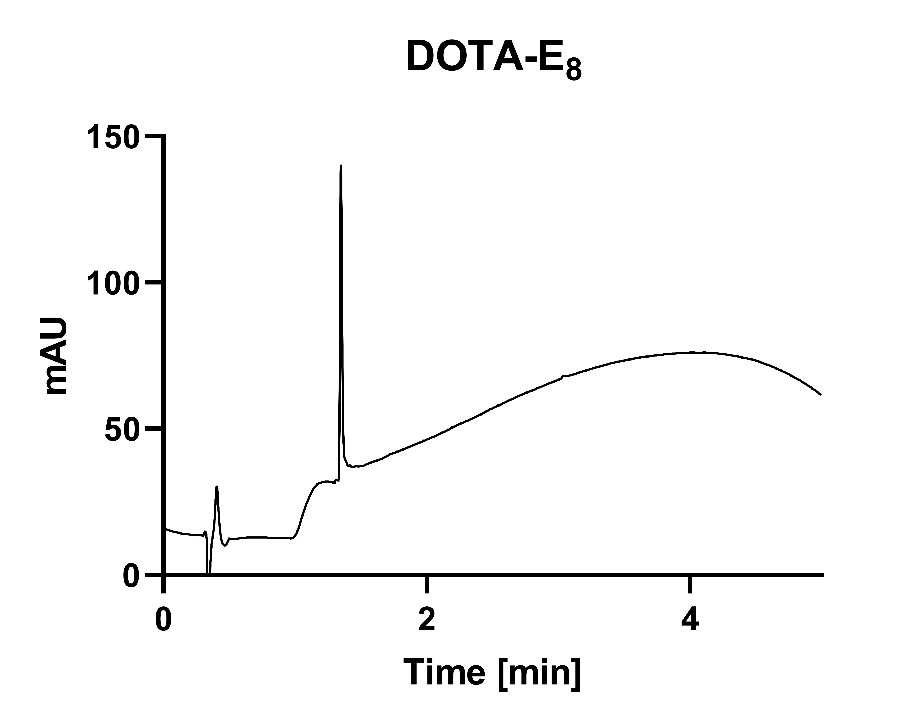
 **Supplemental Figure 20.** RP-HPLC chromatogram of DOTA-E_8_ using conditions C1.

**DOTA-E_8_**

ESI-MS (m/z) for C_56_H_85_N_13_O_31_ [M]: 1435.5474 (calculated); found 718.7788 (718.7810) [M+2H]^2+^.

**DOTA-D_8_**

ESI-MS (m/z) for C_56_H_85_N_13_O_31_ [M]: 1323.4222 (calculated); found 662.6928 (662.7184) [M+2H]^2+^.

**DOTA-(DSS)_6_**

ESI-MS (m/z) for C_76_H_119_N_23_O_49_ [M]: 2137.7566 (calculated); found 1069.7268 (1069.8836) [M+2H]^2+^.

**DOTA-pSE_8_**

ESI-MS (m/z) for C_59_H_91_N_14_O_36_P [M]: 1602.5458 (calculated); found 1603.5625 (1063.5531) [M+H]^+^.

**DOTA-pS_2_E_8_**

ESI-MS (m/z) for C_55_H_80_N_12_O_36_P_2_ [M]: 1769.5442 (calculated); found 885.7605 (885.7799) [M+2H]^2+^; 937.7036 (937.7308) [M-2H+3Na+K]^2+^.

**DOTA-pS_4_E_8_**

ESI-MS (m/z) for C_68_H_109_N_17_O_51_P_4_ [M]: 2103.5409 (calculated); found 1052.7661 (1052.7783) [M+2H]^2+^; 1104.6776 (1104.7291) [M-2H+3Na+K]^2+^.

**DOTA-pS_8_E_8_**

ESI-MS (m/z) for C_80_H_133_N_21_O_71_P_8_ [M]: 2771.5343 (calculated); found 1386.7538 (1386.7750) [M+2H]^2+^; 1438.6981 (1438.7258) [M-2H+3Na+K]^2+^.

**DOTA-MEPE-ASARM**

ESI-MS (m/z) for C_83_H_131_N_27_O_49_ [M]: 2289.8589 (calculated); found 1145.8689 (1145.9373) [M+2H]^2+^.

**DOTA-MEPE-pASARM with three phosphoserines**

ESI-MS (m/z) for C_83_H_134_N_27_O_58_P_3_ [M]: 2579.7579 (calculated); found 1317.7205 (1317.8376) [M-2H+3Na+K]^2+^.

**DOTA-OPN-ASARM**

ESI-MS (m/z) for C_95_H_140_N_30_O_47_ [M]: 2452.987 (calculated); found 1227.4936 (1227.4816) [M+2H]^2+^.

**DOTA-OPN-pASARM with three phosphoserines**

ESI-MS (m/z) for C_95_H_143_N_30_O_56_P_3_ [M]: 2692.8477 (calculated); found 1391.2432 [M-2H+4Na]^2+^.

**DOTA-pS_4_D_8_**

ESI-MS (m/z) for C_60_H_93_N_17_O_51_P_4_ [M]: 1991.4157 (calculated); found 1007.7066 (1007.5461) [M+H+Na]^2+^; 1048.4736 (1048.6665) [M-2H+3Na+K]^2+^.

**DOTA-pS_2_(ESS)_4_**

ESI-MS (m/z) for C_66_H_109_N_19_O_45_P_2_ [M]: 1949.6300 (calculated); found 975.9157 (975.8223) [M+2H]^2+^.

**DOTA-pS_2_**

ESI-MS (m/z) for C_22_H_41_N_7_O_17_P_2_ [M]: 737.2034 (calculated); found 738.0914 (738.2112) [M+H]^+^.

**DOTA-pS_4_**

ESI-MS (m/z) for C_28_H_53_N_9_O_27_P_4_ [M]: 1071.2001 (calculated); found 1072.0421 (1072.2080) [M+H]^+^.

**DOTA-(Cya)_2_E_8_**

ESI-MS (m/z) for C_62_H_95_N_15_O_39_S_2_[M]: 1737.5353 (calculated); found 869.7433 (869.7749) [M+2H]^2+^.

**DOTA-pY_4_E_8_**

ESI-MS (m/z) for C_92_H_125_N_17_O_51_P_4_ [M]: 2407.6661 (calculated); found 1204.7215 (1204.8403) [M+2H]^2+^.
